# Expert Opinion on Optimising Guideline-Directed Medical Therapy and Expanding the Role of Angiotensin Receptor-Neprilysin Inhibitors (ARNIs) for Heart Failure Management in India

**DOI:** 10.7759/cureus.107772

**Published:** 2026-04-26

**Authors:** Bijay Prakash Pandey, Saumitra Ray, Soumitra Kumar, Santanu Guha, Bhupen N Desai, Mahesh K Shah, Srinivas Kudva, Vijay Kumar Chopra, Rajiv Agarwal, Vishal Rastogi, Chandrashekhar K Ponde, Ranjan Kumar Sharma, Dipak Pawar, Gurunath Chavan, Vivek Kolapkar

**Affiliations:** 1 Cardiology, Narayana Superspeciality Hospital, Howrah, IND; 2 Cardiology, Woodlands Hospital, Kolkata, IND; 3 Cardiology, Vivekananda Institute of Medical Sciences, Ramakrishna Mission Seva Pratishthan, Kolkata, IND; 4 Cardiology, Baghbazar Multispeciality Hospitala, Kolkata, IND; 5 Cardiology, Desai Heart Care Clinic, Mumbai, IND; 6 Cardiology, Hinduja Healthcare Limited, Mumbai, IND; 7 Cardiology, Lilavati Hospital & Research Centre, Mumbai, IND; 8 Cardiology, Max Healthcare Institute Limited, New Delhi, IND; 9 Cardiology, Max Smart Superspeciality Hospital, New Delhi, IND; 10 Cardiology, Fortis Escorts Heart Hospital, New Delhi, IND; 11 Cardiology, P. D. Hinduja National Hospital and Medical Research Centre, Mumbai, IND; 12 Cardiology, Manipal Hospitals, Kolkata, IND; 13 Medical Affairs, Lupin Limited, Mumbai, IND

**Keywords:** angiotensin receptor–neprilysin inhibitors, drug utilization, guideline-directed medical therapy, heart failure, sacubitril/valsartan

## Abstract

Strong clinical evidence and clear guideline recommendations support the use of guideline-directed medical therapy (GDMT) - particularly angiotensin receptor-neprilysin inhibitors (ARNIs) - in heart failure (HF) management, yet real-world adoption in India remains limited. Indian registry data indicate that just one quarter of patients leave the hospital with complete GDMT, and less than 5% receive ARNIs, even though robust evidence strongly favors these drugs. Hypotension, renal dysfunction, delayed initiation, and limited familiarity are common barriers that often also hinder optimal use. Sacubitril/valsartan has demonstrated consistent benefits not only in HF with reduced ejection fraction (HFrEF) but also in selected patients with mildly reduced or preserved ejection fraction (HFmrEF/HFpEF), especially women and those with borderline left ventricular ejection fraction, as shown in the Prospective Comparison of ARNI with ACEI to Determine Impact on Global Mortality and Morbidity in Heart Failure (PARADIGM-HF) and Prospective Comparison of ARNI with ARB Global Outcomes in HF With Preserved Ejection Fraction (PARAGON-HF) trials and supported by Indian ARNI PRESERVED study. The Safety, Tolerability and Efficacy of Rapid Up‑Titration of Guideline‑Directed Medical Therapies in Acute Heart Failure (STRONG-HF) trial validated rapid up-titration strategies, demonstrating that early high-intensity GDMT implementation with structured follow-up significantly reduces 180-day mortality and readmissions. Clinical experts recommend early in-hospital initiation of all four pillars of GDMT with flexible, patient-specific sequencing. Approaches such as simultaneous low-dose initiation, careful titration, and close monitoring can help manage tolerability concerns and improve adherence to ARNI therapy. Bridging the gap between guidelines and practice will require systemic interventions, including physician training, patient-centered care models, and devising research strategies such as national HF registries to understand and tailor therapeutic approaches from an Indian perspective.

## Introduction and background

Heart failure (HF), a major global health burden affecting over 64 million people and responsible for 9-10% of cardiovascular deaths annually, impacts an estimated 1.3-4.6 million individuals in India, with an incidence of 0.5-1.8 cases per 1,000 population each year [1,2]. This broad range demonstrates the methodological differences between various epidemiological studies, regional variability in healthcare accessibility and diagnostic process across different regions, and the absence of an integrated national HF surveillance system. In two major Indian registries of HF, HF with reduced ejection fraction (HFrEF) accounted for the majority of cases - 67.5% in the Cardiology Society of India-Kerala Heart Failure Registry (CSI-KHFR; n=7,507) and 65.2% in the National Heart Failure Registry (NHFR; n=10,851) (refer to Appendix), with HF with mildly reduced ejection fraction (HFmrEF) and HF with preserved ejection fraction (HFpEF) comprising the remaining cases of 17.6% vs 22% and 14.9% vs 12.7%, respectively [3]. HF with a high morbidity and mortality rate is reported in India. Around 30-40% of HF patients die within the first year of diagnosis, which is significantly higher compared to 20-25% mortality in the Western registries, such as the European Society of Cardiology Heart Failure Long-Term (ESC-HF-LT) and CHAnge the Management of Patients with Heart Failure (CHAMP-HF), which may be attributed to delayed treatment and insufficient implementation of guideline-directed medical therapy (GDMT) [4]. The Trivandrum Heart Failure Registry revealed that three out of every five patients died during five years of follow-up, with lack of GDMT in HFrEF and frequent readmissions associated with significantly higher five-year mortality [5]. The CSI-KHFR documented an in-hospital and 90-day mortality of 7% and 11.6%, respectively, with acute HF mortality independently associated with GDMT initiation [6]. These findings highlight the urgent need for timely and comprehensive initiation of GDMT in patients with HF, as early implementation is pivotal to improving long-term clinical outcomes.

The financial burden of HF is also substantial, with hospitalizations accounting for approximately 2% of global healthcare expenditures and disproportionately affecting older adults [7,8]. These clinical and economic challenges highlight the urgent need for early, evidence-based interventions. GDMT refers to the evidence-based combination of four drug classes proven to reduce mortality and hospitalisation in HFrEF. Among the renin-angiotensin-aldosterone system (RAAS)-targeting agents within GDMT, angiotensin receptor-neprilysin inhibitors (ARNIs) represent the preferred first-line choice, demonstrating superior outcomes over conventional angiotensin-converting enzyme inhibitor (ACEi) inhibitor therapy in landmark trials. GDMT - comprising pharmacologic agents, such as ACE inhibitors/angiotensin receptor blocker (ARB)/ARNi, beta-blockers (BBs), mineralocorticoid receptor antagonists (MRAs), and sodium-glucose cotransporter 2 inhibitor (SGLT2i) - plays a central role in halting disease progression, reducing hospitalizations, and improving survival and quality of life. However, failure to initiate and optimize GDMT promptly may result in recurrent decompensation, worsening clinical status, and increased mortality risk. High rates of hospitalization and a decline in quality of life, along with increased mortality, are associated with HF. For patients with HF, early implementation of GDMT is essential to lower mortality, prevent readmissions, and enhance quality of life. Survival rates have seen improvement due to the use of GDMT, which includes medications such as SGLT2i, BBs, ACEis, ARBs, ARNIs, and MRAs [9,10].

Despite clinical evidence supporting the benefits of comprehensive GDMT, real-world adherence remains suboptimal, a trend consistent with previous studies [5,6]. The reported GDMT prescription rates, ranging from 25% to 28%, reflect global concerns about the underutilization of evidence-based therapies for HF [11]. This disparity between guideline recommendations and clinical practice highlights the need for guideline-mediated interventions to enhance medication uptake and adherence. The Changes to Health and Attributes of Medication in Patients with Heart Failure (CHAMP-HF) registry in the United States also reported similar challenges, with only 20% of HFrEF patients on target doses of β-blockers, and merely 10% experiencing any up-titration of their therapies over 12 months. Notably, ARNIs were prescribed in only 13% of eligible patients, highlighting a significant gap between guideline recommendations and real-world practice in the use of foundational HF therapies [12]. ARNI is the most underutilized pillar among the four GDMTs, which is prescribed only in 4.8% of eligible HFrEF patients in the National Heart Failure Registry (Appendix), despite exhibiting better outcomes than the conventional ACEis in the Prospective Comparison of ARNI with ACEI to Determine Impact on Global Mortality and Morbidity in Heart Failure (PARADIGM-HF) trial (20% relative risk reduction in cardiovascular death or HF hospitalization; absolute risk reduction 3.2%) [13]. This is due to various factors, such as fragmented insurance coverage, limited access to specialists in rural and urban areas, high medication costs, and a lack of patient follow-up after discharge in India. The epidemiological estimates vary across regions and registries, which are influenced by healthcare access, demographics, and reporting systems in India [1,3]. These findings constitute the specific rationale for this current review and emphasize the urgent need for targeted implementation strategies to improve the uptake of ARNIs.

Given the persistent discrepancies between guideline recommendations and real-world GDMT implementation, there is an urgent need for a structured, evidence-based expert opinion to address the specific barriers affecting ARNI utilization. The key objectives of this review and expert opinion were as follows: (1) to identify real-world barriers to GDMT and ARNI underutilization; (2) to compare regional implementation challenges in India to identify adaptable solutions; and (3) to propose practical strategies, including policy recommendations, to improve GDMT adherence and timely uptitration of ARNI.

Methodology

This is a narrative review of the existing evidence combined with the expert consensus opinion of Indian cardiologists. Databases such as PubMed, MEDLINE, and Cochrane Database of Systematic Reviews were searched using terms such as "heart failure", "GDMT", "ARNI", "sacubitril/valsartan", and "India". A comprehensive assessment of guideline documents and Indian registry publications was also performed for this analysis. A structured consultative process involving Indian cardiologists with extensive clinical expertise in the management of HF was used for expert opinions.

The presented evidence-based indications regarding ARNI in HFrEF (supported by level A randomized trial evidence) are stated independently from the emerging subgroup data in HFmrEF and HFpEF throughout this review. No novel statistical tests or meta-analysis were performed, and all effect estimates mentioned were derived from individual published trials, which are cited accordingly. This review and professional opinion would contribute to the gap between clinical studies and clinical practice, which would also assist in the enhancement of patient outcomes.

## Review

The role of GDMT in HF

The management of HFrEF has evolved remarkably over the last four decades - from symptomatic relief to precise, evidence-driven, multidrug modulation of maladaptive neurohormonal pathways. The therapeutic paradigm now rests on four foundational drug classes that synergistically address the RAAS, the sympathetic nervous system (SNS), myocardial fibrosis, and systemic metabolic dysregulation. Collectively termed GDMT, these therapies form the cornerstone of HFrEF management, translating molecular insight into measurable survival gains.

Evolution of therapeutic evidence

The introduction of ACEis in the 1980s was a pivotal moment that changed the natural history of HFrEF. The Cooperative North Scandinavian Enalapril Survival Study (CONSENSUS) and Studies of Left Ventricular Dysfunction (SOLVD) trials [8,14,15] demonstrated striking reductions in mortality and hospitalization through afterload reduction and attenuation of maladaptive ventricular remodelling. By interrupting the RAAS cascade, ACEis reduced vasoconstriction, sodium retention, and neurohormonal overdrive, thereby mitigating progression to end-stage HF. Their benefits were dose-dependent; higher enalapril doses in the SOLVD trial produced greater mortality reductions among deaths due to progressive HF.

In ACEi-intolerant patients, ARBs were validated through the Candesartan in Heart Failure: Assessment of Reduction in Mortality and Morbidity - Alternative (CHARM-Alternative) trial [16], which showed comparable survival benefits by selectively blocking the angiotensin II type 1 receptor. Together, ACEi and ARB therapy established the first line of disease-modifying pharmacotherapy in HFrEF.

The subsequent addition of BBs revolutionized management by addressing sympathetic overactivation - a compensatory but deleterious mechanism in HF progression. Trials such as Cardiac Insufficiency Bisoprolol Study II (CIBIS-II), Metoprolol CR/XL Randomized Intervention Trial in Congestive Heart Failure (MERIT-HF), and Carvedilol Prospective Randomized Cumulative Survival Trial (COPERNICUS) [14,17] consistently demonstrated 30-35% reductions in mortality and hospitalizations. β-blockade improved left-ventricular ejection fraction (LVEF), reversed remodelling, and reduced sudden cardiac death. The Markers of Cardiac Damage and Heart Failure (MOCHA) trial [18] elegantly established a dose-response relationship between carvedilol-controlled release/extended release (CR/XL) and improvements in ejection fraction and survival, highlighting the importance of dose titration to achieve maximal benefit (see Table 1 for a summary of landmark trials, relative risk reduction (RRR), absolute risk reduction (ARR), and safety considerations for all four GDMT pillars). MRAs emerged as the third therapeutic pillar when the Randomized Aldactone Evaluation Study (RALES) and Eplerenone in Mild Patients Hospitalization and Survival Study in Heart Failure (EMPHASIS-HF) trials confirmed mortality and hospitalization benefits across the severity spectrum. By inhibiting aldosterone-mediated sodium retention and myocardial fibrosis, MRAs enhanced survival while improving ventricular compliance and diastolic relaxation. Together, ACEi/ARB + BBs + MRA therapy reduced mortality by nearly half compared with conventional care, redefining the long-term prognosis of HFrEF (see Table 1) [19,20].

**Table 1:** Summary of landmark trials for the four pillars of GDMT in HFrEF ACEi = angiotensin-converting enzyme inhibitor; ARB = angiotensin receptor blocker; ARNI = angiotensin receptor-neprilysin inhibitor; ARR = absolute risk reduction; BD = twice daily; BP = blood pressure; CV = cardiovascular; DKA = diabetic ketoacidosis; DM = diabetes mellitus; eGFR = estimated glomerular filtration rate; GLP-1RA = glucagon-like peptide-1 receptor agonist; GWTG-HF = Get With the Guidelines-Heart Failure; HF = heart failure; HFmrEF = heart failure with mildly reduced ejection fraction; HFpEF = heart failure with preserved ejection fraction; HFrEF = heart failure with reduced ejection fraction; HR = hazard ratio; K+ = serum potassium; KCCQ-CSS = Kansas City Cardiomyopathy Questionnaire Clinical Summary Score; LMIC = low- and middle-income countries; LVEF = left ventricular ejection fraction; MRA = mineralocorticoid receptor antagonist; NT-proBNP = N-terminal pro-B-type natriuretic peptide; NNT = number needed to treat; NYHA = New York Heart Association; QoL = quality of life; RR = rate ratio; RRR = relative risk reduction; SBP = systolic blood pressure; SGLT2i = sodium-glucose cotransporter-2 inhibitor; SR = strongly recommend; T2DM = type 2 diabetes mellitus; TTR = transthyretin; 6MWT = 6-minute walk test

GDMT Pillar	Drug Class/Agent	Key Trials	Primary Endpoint	Relative Risk Reduction (RRR)	Absolute Risk Reduction (ARR)	Principal Safety Considerations
PILLAR 1 - RAAS Inhibition: ACE Inhibitors/Angiotensin Receptor Blockers/ARNI						
Pillar 1 ACE Inhibitor	Enalapril	CONSENSUS [15]	Composite: all-cause mortality in severe HFrEF (NYHA IV)	40% ↓ Mortality	~14% (6-month data)	Hypotension, renal impairment, hyperkalaemia, cough, angioedema (rare)
Pillar 1 ACE Inhibitor	Enalapril	SOLVD [15]	All-cause mortality in HFrEF (LVEF ≤35%)	16% ↓ Mortality	4.5% (3.5 yr follow-up)	Hypotension, renal dysfunction, hyperkalaemia, dry cough (10-15%), angioedema
Pillar 1 ARB	Candesartan	CHARM-Alternative [16]	CV death or HF hospitalisation in ACEi-intolerant HFrEF	23% ↓ CV death/HF hosp.	7% (3-year data)	Hypotension, renal impairment, hyperkalaemia; lower cough vs ACEi; angioedema rare
Pillar 1 ARNI	Sacubitril/Valsartan	PARADIGM-HF [9]	CV death or first HF hospitalisation vs enalapril in HFrEF (LVEF ≤35%, NYHA II-IV)	20% ↓ CV death/HF hosp. 16% ↓ All-cause mortality 21% ↓ HF hosp.	4.7% (composite) 2.8% (all-cause mortality) (27-month follow-up) NNT = 21 (composite endpoint; 27 months); NNT = 32 (CV death; 27 months)	Hypotension (18% vs 12%), renal impairment, hyperkalaemia; less cough than ACEi; angioedema risk - must not combine with ACEi (36-hr washout required)
Pillar 1 ARNI	Sacubitril/Valsartan	PIONEER-HF [9]	NT-proBNP reduction from baseline at 4 & 8 weeks: in-hospital initiation in acute decompensated HFrEF	29% greater ↓ NT-proBNP vs enalapril	Biomarker endpoint (no ARR reported)	Symptomatic hypotension (9.9% vs 6.9%), renal impairment, hyperkalaemia; no excess worsening renal function or hyperkalaemia vs enalapril
Pillar 1 ARNI	Sacubitril/Valsartan	TRANSITION [9]	Proportion achieving target dose (97/103 mg BD) at 10 weeks: pre-discharge vs post-discharge initiation	Target dose achieved in 45-52% at 10 weeks	Feasibility trial (no ARR)	Hypotension most common reason for dose interruption; renal dysfunction and hyperkalaemia in <5%; overall well-tolerated in the post-discharge setting
Pillar 1 ARNI	Sacubitril/Valsartan	TITRATION [21]	Proportion achieving target dose (97/103 mg BD) at 12 weeks: condensed vs conservative up-titration	Conservative arm: 76% reached target dose vs 84% condensed	Tolerability trial (no ARR)	Symptomatic hypotension more frequent with rapid titration; conservative titration recommended in patients with SBP <110 mmHg or renal impairment
Pillar 1 ARNI (Indian HFrEF Real-World)	Sacubitril/Valsartan	SAVE Study [22]	LVEF improvement and NT-proBNP reduction at 3 months; symptomatic improvement (breathlessness, oedema, palpitations) in HFrEF (NYHA I-IV) in an Indian tertiary care setting (N=60; retrospective cohort)	23% improvement in mean LVEF (34-42%; p<0.0001); NT-proBNP reduced from 1220.5 to 118.3 pg/mL (p<0.0001); NYHA class improvement (66.7% in class I at 3 months); real-world Indian HFrEF cohort	Real-world observational study; no placebo comparator; 3-month follow-up (no ARR calculable)	Well tolerated in the Indian HFrEF population; hypotension reported in <5% patients (none discontinued therapy); no major eGFR change at 3 months; 60% patients had both T2DM and hypertension as comorbidities - comorbidities had no impact on treatment efficacy; most patients received 50 mg BD (70%) or 100 mg BD (25%)
PILLAR 2 - Sympathetic Nervous System Blockade: Beta-Blockers						
Pillar 2 Beta-Blocker	Bisoprolol	CIBIS-II [14,17]	All-cause mortality in symptomatic HFrEF (LVEF <35%, NYHA III-IV)	34% ↓ All-cause mortality	5.5% (1.3-yr median follow-up)	Bradycardia, hypotension, worsening HF on initiation, fatigue, bronchospasm (avoid in COPD/asthma); do not initiate in decompensated HF
Pillar 2 Beta-Blocker	Metoprolol CR/XL	MERIT-HF [17]	All-cause mortality in symptomatic HFrEF (LVEF <40%, NYHA II-IV)	34% ↓ All-cause mortality	5.0% (1-yr follow-up)	Bradycardia, hypotension, fatigue; risk of decompensation if initiated or up-titrated too rapidly; cardioselective (lower bronchospasm risk)
Pillar 2 Beta-Blocker	Carvedilol	COPERNICUS [14]	All-cause mortality in severe HFrEF (LVEF <25%, NYHA III-IV)	35% ↓ All-cause mortality	5.1% (10.4-mo follow-up)	Hypotension (especially on initiation due to α1 blockade), bradycardia, worsening HF, dizziness; must start low and titrate slowly
Pillar 2 Beta-Blocker	Carvedilol CR/XL	MOCHA [18]	Dose-response: LVEF improvement and mortality reduction across carvedilol dose tiers (6.25/12.5/25 mg BD)	Dose-dependent: up to 31% ↓ Mortality	~5% (6-mo follow-up; dose-dependent)	Dose-dependent hypotension and bradycardia; underlines the importance of up-titration to the maximum tolerated dose rather than a fixed low dose
PILLAR 3 - Aldosterone Antagonism: Mineralocorticoid Receptor Antagonists (MRAs)						
Pillar 3 MRA	Spironolactone	RALES [19]	All-cause mortality in severe HFrEF (LVEF <35%, NYHA III-IV) on background ACEi + loop diuretic	30% ↓ All-cause mortality	11.4% (24-mo follow-up; trial stopped early)	Hyperkalaemia (risk highest with eGFR <45 or K+ >5.0), gynaecomastia (10%), renal impairment; avoid if K+ >5.0 or eGFR <30
Pillar 3 MRA	Eplerenone	EMPHASIS-HF [20]	CV death or HF hospitalisation in mild HFrEF (LVEF ≤35%, NYHA II) on background ACEi/ARB + β-blocker	37% ↓ CV death/HF hosp. 24% ↓ All-cause mortality	7.7% (composite) 3.4% (mortality) (21-month median follow-up)	Hyperkalaemia (serious in 2.5% vs 1.9% placebo), renal impairment; eplerenone has no gynaecomastia; monitor K+ and renal function at 1, 4, 8, 12 weeks, then every 4 months
Pillar 3 MRA (HFpEF/HFmrEF)	Finerenone	FINEARTS-HF Trial [23]	CV death or total HF events in HFpEF/HFmrEF (LVEF ≥40%)	~16% ↓ CV death or worsening HF events (HR 0.84, 95% CI: 0.74-0.95)	Statistically significant reduction (p=0.007); rate ratio 0.84 (95% CI: 0.74-0.95); absolute event reduction not separately quantified in this table	Hyperkalaemia (more common than placebo; monitor K+ and eGFR regularly); lower gynaecomastia risk vs spironolactone; avoid if K+ >5.0 mEq/L or eGFR <25 mL/min
PILLAR 4 - Cardiometabolic Protection: SGLT2 Inhibitors						
Pillar 4 SGLT2i	Dapagliflozin	DAPA-HF [8]	CV death, worsening HF or urgent HF visit in HFrEF (LVEF ≤40%) - diabetic & non-diabetic	26% ↓ Composite	5.0% (18.2-mo median follow-up)	Genital mycotic infections, volume depletion/hypotension, DKA (rare in T2DM, very rare without DM); avoid if eGFR <25; no dose titration required
Pillar 4 SGLT2i	Empagliflozin	EMPEROR-Reduced [24]	CV death or HF hospitalisation in HFrEF (LVEF <40%) - diabetic & non-diabetic	25% ↓ CV death/HF hosp.	5.3% (16-mo median follow-up)	Genital mycotic infections, urinary tract infections, volume depletion, DKA (rare); renal function: modest eGFR decline on initiation but slower long-term eGFR loss; avoid if eGFR <20
Pillar 4 SGLT2i (HFpEF/HFmrEF)	Empagliflozin	EMPEROR-Preserved trial [25]	Composite CV death, HF hospitalisation, or emergent/urgent HF visit in HFpEF/HFmrEF (LVEF ≥40%) - diabetic and non-diabetic	23% ↓ composite endpoint (CV death/HF hosp/urgent HF). Critically: statistical significance achieved as early as 18 days post-randomisation - confirming even a 2-3 week delay in SGLT2i initiation carries a significant opportunity cost. Benefits are consistent regardless of background, HF therapy, and polypharmacy.	Statistically significant ARR (event-rate based; primary composite endpoint); EF-independent benefit	Genital mycotic infections, urinary tract infections, volume depletion (similar to HFrEF); DKA rare; avoid if eGFR <20. Benefits are fully additive regardless of background SGLT2i. Note: benefits are consistent irrespective of the number of background medications - directly counters polypharmacy hesitancy concern raised in a study [25].
All 4 Pillars (GDMT)	All GDMT agents (rapid up-titration)	STRONG-HF [26]	180-day all-cause mortality or HF readmission: high-intensity GDMT up-titration within 2 weeks post-discharge vs usual care	34% ↓ 180-day HF readmission or death (STRONG-HF). GWTG-HF registry [27]; n=33,036 newly diagnosed HFrEF): in-hospital quadruple GDMT initiation can yield up to 25% absolute risk reduction in 1-year mortality (NNT=4); >80% of newly diagnosed HFrEF eligible for quadruple GDMT; only 15% prescribed quadruple GDMT and 42% triple therapy in real-world practice	8.1% (180-day follow-up; trial stopped early)	Worsening renal function, hypotension, and hyperkalaemia more frequent in the high-intensity arm (all manageable); this highlights the need for structured follow-up at 1-2 weeks post-discharge. In-hospital initiation of BB, ARNI, and MRA associated with a threefold increase in post-discharge medication fills. NATRIUM-HF study: sacubitril/valsartan initiation in stable HF improved diuretic response and reduced sodium retention
ARNI IN HFpEF/HFmrEF - Expanding Indications (Supplementary Evidence)						
ARNI (HFpEF/HFmrEF)	Sacubitril/Valsartan	PARAGON-HF [28,29]	CV death or total HF hospitalisations in HFpEF (LVEF ≥45%, n=4,822)	RR 0.87 (p=0.06); 27% ↓ HF hosp. in women (P interaction =0.017). 4 RCTs: PARAMOUNT, PARAGON-HF, PARALLAX, PARAGLIDE-HF; n=6,737): pooled composite HF hospitalisation + CV death RR 0.86 (95% CI: 0.75-0.99, p=0.04); KCCQ-CSS MD +1.13 (95% CI: 0.15-2.11, p=0.024); NYHA class improvement OR 1.32 (95% CI: 1.10-1.59, p=0.002); no significant benefit for CV death (RR: 0.92, 95% CI: 0.77-1.10, p=0.38) or all-cause mortality (RR: 0.96, 95% CI: 0.85-1.10, p=0.56)	Not statistically significant overall; ~1.0 event/patient/yr vs 1.15. Meta-analysis [30]: significant relative benefit confirmed (composite RR 0.86); no statistically significant ARR for CV death or all-cause mortality endpoints across pooled RCTs	Hypotension occurred in 13.3% overall (637/4,796 patients); 16% sacubitril/valsartan vs 11% valsartan; p<0.001 [31]; renal impairment, hyperkalaemia; angioedema rare; similar tolerability to HFrEF cohort; prefer initiation at lower doses in elderly women. LVEF ≥60% independently predicts greater hypotension risk AND reduced clinical efficacy with sacubitril/valsartan - benefit/risk ratio favours sacubitril/valsartan only in LVEF below normal (<60%); in LVEF <60%, sacubitril/valsartan prevents ~3 primary events per 1 extra hypotensive episode vs valsartan; in LVEF ≥60%, 0 primary events prevented but ~3 extra hypotensive episodes per 100 patients. Independent predictor: LVEF per 5% increase HR 1.11 (95% CI: 1.06-1.17; p<0.001). P interaction for LVEF × treatment on hypotension risk = 0.019. Post-hypotension: adjusted RR 1.63 (95% CI: 1.27-2.09) for CV death/HF hospitalisation; HR 1.62 (95% CI: 1.28-2.05) for all-cause mortality; drug discontinuation HR 1.57 (95% CI: 1.31-1.87; p<0.001). The treatment benefits of sacubitril/valsartan remained consistent irrespective of the occurrence. Meta-analysis [30] (4 RCTs, n=6,737): pooled hypotension OR 1.67 (95% CI: 1.27-2.19, p<0.0001) vs valsartan; no significant excess hyperkalaemia (OR: 0.90, 95% CI: 0.78-1.03, p=0.124) or worsening renal function (OR: 0.80, 95% CI: 0.55-1.16, p=0.241) - confirming hypotension as the primary safety concern while renal function and potassium are not disproportionately worsened
ARNI (HFpEF/HFmrEF)	Sacubitril/Valsartan	PARAGLIDE-HF + PARAGON-HF (pooled) [32]	CV death or total HF hospitalisations in HFmrEF/HFpEF (LVEF >40%); pre-specified participant-level pooled analysis	~16% ↓ Composite (HF hosp. driven; no mortality benefit). Pooled RR 0.86 (95% CI: 0.75-0.99, p=0.04) for composite HF hosp + CV death across 4 RCTs (n=6,737); NYHA class improvement OR 1.32 (p=0.002); KCCQ-CSS MD +1.13 (p=0.024); no CV death or all-cause mortality benefit confirmed	Mainly hospitalisation-driven benefit; mortality neutral	As per PARAGON-HF, benefits are greatest in LVEF ≤57% and in women; initiate at low dose; monitor BP and renal function closely. Per Zaman et al. [31] Sacubitril/valsartan benefit/risk ratio is most favourable in LVEF below normal (<60%); LVEF ≥60% is associated with higher treatment-related hypotension risk and no demonstrable reduction in primary HF events - reinforcing LVEF <60% as the key threshold for initiating sacubitril/valsartan in HFmrEF/HFpEF. Meta-analysis [30]: pooled hypotension OR 1.67 (95% CI: 1.27-2.19, p<0.0001); no significant excess hyperkalaemia (OR 0.90) or worsening renal function (OR 0.80) vs valsartan - clinicians should counsel patients that hypotension is the primary adverse effect to monitor, while renal function and potassium are not disproportionately worsened
ARNI (Indian HFpEF Cohort)	Sacubitril/Valsartan	ARNI-PRESERVED (India) [33]	Safety and effectiveness at 6 months: NYHA class, exercise tolerance and quality of life in Indian HFpEF patients	Significant NYHA class improvement at 6 months	Real-world observational; no placebo comparator	Well tolerated in the Indian HFpEF cohort; hypotension is manageable with low starting doses; supports feasibility in resource-limited settings
GLP-1RA (HFpEF with Obesity)	Semaglutide/Tirzepatide	STEP-HFpEF, STEP-HFpEF DM (pooled), and SUMMIT [23]	Symptoms/QoL (KCCQ-CSS), 6MWT improvement, and worsening HF events in obese HFpEF patients	Semaglutide: ~7 pt ↑ KCCQ-CSS; ~20 m ↑ 6MWT vs placebo Tirzepatide (SUMMIT): 38% ↓ worsening HF events or CV death	Tirzepatide: HR 0.62 (95% CI: 0.41-0.95) for composite worsening HF/CV death; weight reduction ~15%	GI side effects (nausea, vomiting, diarrhoea - dose-dependent); generally well tolerated; avoid in history of pancreatitis or medullary thyroid carcinoma; iCARDIO 2025 strongly recommends GLP-1RA (semaglutide/tirzepatide) in obese HFpEF patients (SR)
UPDATED GUIDELINE	iCARDIO Alliance Global Implementation Guidelines on Heart Failure 2025	iCARDIO Alliance 2025 [23]	Global implementation framework for HF management incorporating novel therapies, resource-limited settings (SR/R/Su/DND grading); covers HFrEF, HFpEF, cardiomyopathies	Strongly Recommends (SR): SGLT2i, ARNI, BB, MRA for HFrEF; SGLT2i + MRA for HFpEF; GLP-1RA for obese HFpEF; tafamidis/acoramidis/vutrisiran for TTR amyloidosis	FRESH-UP trial [34]: fluid restriction to ~2 L/day recommended (R) in chronic HF - provides a specific quantitative threshold for fluid management guidance	(1) FAIR-HF2: IV ferric carboxymaltose (SR) for iron deficiency in HFrEF - reduces HF hospitalisation/CV death; monitor ferritin and TSAT. (2) HELIOS-B/vutrisiran: vutrisiran (SR) for TTR amyloid cardiomyopathy - reduced all-cause mortality + CV events vs placebo. (3) FRESH-UP: fluid restriction to ~2 L/day (R) in chronic HF is safe and reduces congestion without adverse renal or electrolyte effects

The fourth pillar - ARNI and beyond

The fourth major advance came with ARNIs, combining valsartan with sacubitril to deliver dual RAAS suppression and augmentation of natriuretic peptides. The PARADIGM-HF trial [9] proved that this mechanistic synergy translated into clinically superior outcomes: a 20% RRR (ARR: 3.2%) in the composite of cardiovascular death or HF hospitalization, and 16% lower all-cause mortality (ARR: 2.8%), yielding a number needed to treat (NNT) of 21 over 27 months to prevent one primary composite event, and 32 to prevent one cardiovascular death and improved quality-of-life scores versus enalapril. ARNI therapy thus became the preferred RAAS-modulating strategy for chronic HFrEF (Table 1).

The STRONG-HF trial further advanced this framework by demonstrating that rapid up-titration of all GDMT components within two weeks of discharge, accompanied by structured follow-up, achieved a significant reduction in 180-day all-cause mortality and HF readmissions compared with usual care. This pivotal study validated a shift from the traditional cautious, stepwise approach to early, high-intensity GDMT implementation, highlighting that therapeutic velocity is as critical as therapeutic selection [26]. However, the feasibility of such intense up-titration regimens in low-resource Indian hospitals should be considered carefully while adapting to the local context because of limited availability of specialists, constrained follow-up infrastructure, and substantial high costs, thereby affecting the clinical reassessment. Concurrently, the advent of SGLT2i - dapagliflozin and empagliflozin - has expanded the HF armamentarium beyond neurohormonal modulation. The Dapagliflozin and Prevention of Adverse Outcomes in Heart Failure (DAPA-HF) and Empagliflozin Outcome Trial in Patients with Chronic Heart Failure and a Reduced Ejection Fraction (EMPEROR-Reduced) trials [8,24] showed robust 25-30% reductions in the composite of cardiovascular death or HF hospitalization, irrespective of diabetic status. These agents improve myocardial energetics, promote osmotic diuresis, reduce afterload, and confer renal protection without necessitating dose titration - making them an ideal adjunct to the established triad of ARNIs/ACEis/ARBs, BBs, and MRAs (Table 1).

Mechanistic convergence and clinical synergy

The four pillars of GDMT act on complementary yet interconnected axes of HF pathophysiology. Rather than acting through a single pathway, the four pillars of GDMT provide complementary prognostic and symptomatic benefits, collectively reducing mortality and HF hospitalization while improving quality of life. ACEis/ARBs/ARNIs suppress maladaptive RAAS activation, BBs blunt the excessive sympathetic drive, MRAs prevent aldosterone-induced fibrosis, and SGLT2is improve cardiometabolic efficiency and vascular function. Collectively, they remodel the failing myocardium, enhance natriuretic peptide signalling, stabilize hemodynamics, and preserve renal function. Evidence from post-hoc and meta-analytic data supports the additive and independent benefits of each class, even when layered sequentially, and across a broad range of patient phenotypes. Reduction in the mortality rate of 25-30% was reported for SGLT2is, representing RRR in cardiovascular death or HF hospitalization, as an outcome of dedicated HFrEF trials (DAPA-HF, EMPEROR-Reduced); however, these data should be interpreted cautiously when extrapolated to broader HF phenotypes. Various safety issues, such as hypotension (especially when used in combination with ARNI and diuretics), transient decline in renal function, and hyperkalemia with MRA, should be weighed against the benefits of rapid multi-pillar adoption, especially in patients with hemodynamic issues. In India, the observed GDMT uptake and outcomes from the real world registries data are significantly lower than the data from the clinical trials, which may be due to the compounding effects of delayed initiation, suboptimal dosing, and limited follow-up [8,10,19,20,24,35]. Hence, the emphasis in contemporary practice has shifted from monotherapy optimisation to complete, rapid, multi-pillar implementation (Table 1).

From guidelines to practice: The 4×4 approach

**Figure 1:**
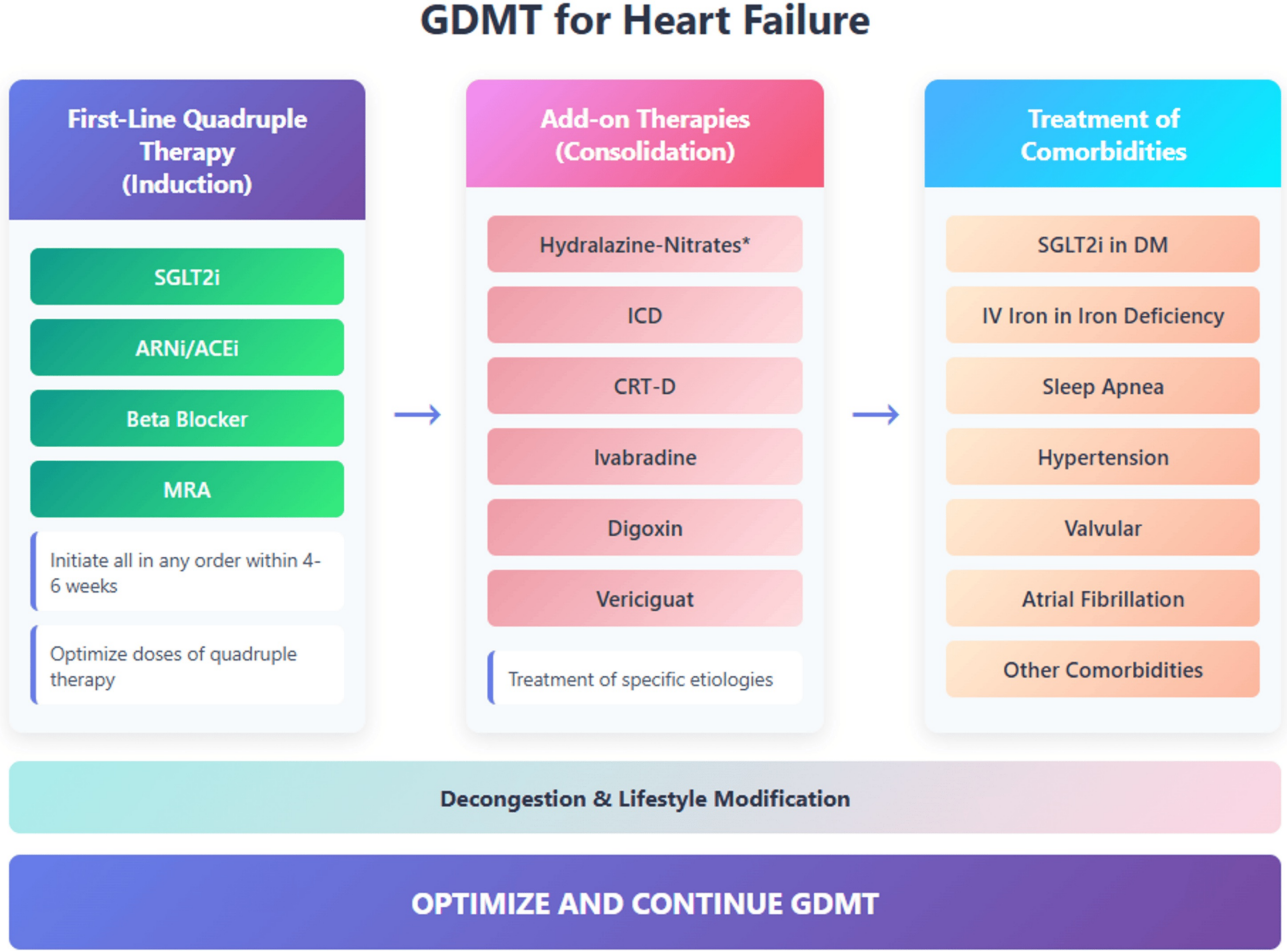
Strategies for the initiation and optimization of GDMT in heart failure with reduced ejection fraction (HFrEF) The figure illustrates the recommended approach to first-line quadruple therapy (induction phase), add-on therapies (consolidation phase), and treatment of comorbidities, with the goal of reducing mortality, hospitalizations, and improving quality of life. ACEi = angiotensin-converting enzyme inhibitor; ARB = angiotensin receptor blocker; ARNI = angiotensin receptor-neprilysin inhibitor; MRA = mineralocorticoid receptor antagonist; SGLT2i = sodium-glucose cotransporter-2 inhibitor Adapted from contemporary guideline recommendations [35,39,40].

Current global guidelines advocate for the early initiation of all four classes within weeks of diagnosis. The American Heart Association (AHA), American College of Cardiology (ACC), Heart Failure Society of America (HFSA) 2022, and European Society of Cardiology (ESC) 2021 recommendations promote the "4×4" strategy - introducing BB and SGLT2i immediately upon stabilization, followed by an ACEi/ARB or, preferably, ARNI and an MRA by week four [35]. The rationale is compelling: each day of delay in initiating or optimizing GDMT exposes patients to continued neurohormonal toxicity and preventable morbidity. The STRONG-HF findings provide strong empirical support for this high-intensity sequencing model [26,36], demonstrating improved NT-proBNP profiles, lower 180-day event rates, and better patient-reported outcomes with structured early follow-up. Strategies for initiation and optimization of GDMT in HF are shown in Figure 1. Despite these clear recommendations, real-world data reveal a persistent gap between evidence and implementation. Registries such as CHAMP-HF, CHECK-HF, ASIAN-HF, ESC-HF-LT, and Indian datasets [11,37,38] consistently show that only about one-quarter of HFrEF patients receive all foundational drugs at discharge, and even fewer achieve target doses. The reasons are multifactorial - therapeutic inertia, clinician unfamiliarity with rapid titration, hypotension or renal concerns, and systemic barriers, such as limited follow-up infrastructure and medication cost. In low- and middle-income settings, the challenge is compounded by restricted access to newer drugs such as ARNI and SGLT2is. See Table 2 for a phased, week-by-week GDMT initiation schedule with India-specific adaptations.

**Table 2:** Guideline-directed medical therapy for heart failure: A phased approach to initiation, dose escalation, and monitoring with India-specific adaptations SGLT2i = sodium-glucose cotransporter-2 inhibitor; ARNI = angiotensin receptor–neprilysin inhibitor; MRA = mineralocorticoid receptor antagonist; BB = beta-blocker; GLP-1RA = glucagon-like peptide-1 receptor agonist; SBP = systolic blood pressure; eGFR = estimated glomerular filtration rate; K+ = serum potassium; BD = twice daily; OD = once daily; IHD = ischaemic heart disease; CCB = calcium channel blocker; ICD = implantable cardioverter-defibrillator; CRT-D = cardiac resynchronisation therapy with defibrillator; HFSI = Heart Failure Society of India; PMJAY = Pradhan Mantri Jan Arogya Yojana; NT-proBNP = N-terminal pro-B-type natriuretic peptide; DM = diabetes mellitus; HTN = hypertension; AF = atrial fibrillation; TTR = transthyretin; T2DM = type 2 diabetes mellitus; CKD = chronic kidney disease; LMIC = low- and middle-income countries; SR = strongly recommend; R = recommend; Su = suggest

Phase	Drug Class	Starting Dose (Example)	Target Dose (Example)	Timing/Escalation	India-Specific Considerations
Phase 1 - Induction (Hospitalisation/Discharge)					
Phase 1 - Induction (Hospitalisation/Discharge)	SGLT2 Inhibitor (Dapagliflozin/Empagliflozin)	-	Fixed dose; no up-titration required	Initiate at or before discharge; week 0-1	Generic formulations available; cost-effective. Avoid dapagliflozin if eGFR <25 mL/min; avoid empagliflozin if eGFR <20 mL/min (HF indication) (per prescribing information and ESC 2021 HF Guidelines). Preferred early, given no titration burden. iCARDIO 2025 strongly recommends (SR) SGLT2i in all HFrEF patients and for HFpEF, further supporting early initiation in Indian clinical practice regardless of diabetes status. Rapid or simultaneous initiation of all 4 GDMT pillars - including SGLT2i - at the time of HF diagnosis or hospitalisation is supported by the GWTG-HF registry [27] (n=33,036): >80% of newly diagnosed HFrEF patients are eligible for quadruple GDMT, yet only 15% are prescribed quadruple therapy in real-world practice; in-hospital quadruple GDMT initiation can yield up to 25% absolute risk reduction in 1-year mortality (NNT=4). In EMPEROR-Preserved, SGLT2i statistical significance was achieved as early as 18 days post-randomisation - confirming that even brief delays carry significant opportunity cost. In-hospital initiation of GDMT is also associated with a >3-fold increase in post-discharge medication adherence vs deferred initiation [41]. Clinicians should not allow polypharmacy concerns to delay initiation: GDMT benefits are consistent regardless of background medication count [42].
	ARNI (Sacubitril/Valsartan)	24/26 mg BD (or 49/51 mg BD if haemodynamically stable with SBP ≥100 mmHg, regardless of prior RAAS therapy)	97/103 mg BD	Start in-hospital if SBP ≥100 mmHg; double dose every 2-4 weeks	Begin at the lowest dose (24/26 mg) if SBP is 90-100 mmHg. eGFR <30 mL/min is a relative contraindication; if clinical judgement favours initiation, use only 24/26 mg BD with intensive renal monitoring (per prescribing guidance). Avoid if K+ >5.4. Out-of-pocket cost is a key barrier; advocate for insurance/PMJAY coverage. iCARDIO 2025 [23] recommends (R) ARNI as first-line RAAS inhibitor in HFrEF; also recommends (R) ARNI in HFpEF. In resource-limited settings, ACEi/ARB remain acceptable alternatives per iCARDIO economic adjustment recommendations.
	Beta-Blocker (Carvedilol/Bisoprolol/Metoprolol XL)	-	Carvedilol 25 mg BD; bisoprolol 10 mg OD; metoprolol XL 200 mg OD	Start once euvolaemic; double dose every 2 weeks (weeks 0-4)	Generic beta-blockers widely available and affordable. Avoid initiating during acute decompensation. Carvedilol preferred in diabetics for alpha-blockade benefit). iCARDIO 2025 strongly recommends (SR) beta-blockers in all HFrEF patients; underscores that carvedilol, bisoprolol, and metoprolol succinate are evidence-based choices [23].
	MRA (Spironolactone/Eplerenone)	Spironolactone 25 mg OD; Eplerenone 25 mg OD	Spironolactone 50 mg OD; eplerenone 25-50 mg OD (25 mg if eGFR <50 mL/min; 50 mg if eGFR ≥50 mL/min)	Initiate with other GDMT; up-titrate at 4 weeks if K+ <5.0 & eGFR stable	Spironolactone preferred (lower cost). Monitor renal function and K+ at 1-2 weeks post-initiation. Avoid initiation if K+ >5.0 or eGFR <30. Eplerenone dose is eGFR-dependent (EMPHASIS-HF strata [20]). iCARDIO 2025 strongly recommends (SR) MRA in HFrEF; also recommends (R) MRA including finerenone (R) for HFpEF - particularly important for Indian patients with coexisting T2DM and CKD [23].
Phase 1/Phase 3 (HFpEF)	Finerenone (Non-steroidal MRA)	10 mg OD (starting dose; increase to 20 mg OD if tolerated and K+ ≤4.8 mEq/L)	20 mg OD (target dose per FINEARTS-HF; adjust to 10 mg OD if eGFR 25-60 mL/min)	Initiate in HFpEF/HFmrEF (LVEF ≥40%); check K+ and eGFR before and 4 weeks after initiation	FINEARTS-HF demonstrated ~16% reduction in CV death or worsening HF events in HFpEF/HFmrEF. iCARDIO 2025 grades finerenone (R) above spironolactone (Su) for HFpEF. Finerenone has lower hyperkalaemia and gynaecomastia risk vs spironolactone. Particularly relevant for Indian HFpEF patients with T2DM/CKD. Where finerenone is unavailable due to limited access in India, spironolactone (Su) remains an alternative per iCARDIO 2025 economic adjustment guidance [23].
Phase 2 - Consolidation (Weeks 4-6 post-discharge)					
Phase 2 Consolidation (Weeks 4-6 Post-Discharge)	Up-titration of all 4 pillars	-	Maximum tolerated dose for each agent	-	Nurse-led HF clinics or teleconsultation can facilitate dose escalation in resource-limited settings. Use simplified escalation algorithms. iCARDIO 2025 supports non-invasive home tele-monitoring (Su) to guide management and reduce hospitalisations in resource-limited settings [23].
	Add-on Therapies if indicated: Ivabradine, Digoxin, Vericiguat, Hydralazine-Nitrates, ICD/CRT-D		-	-	Device therapy (ICD/CRT-D) access is limited to tertiary centres. Prioritise pharmacological optimisation first in primary/secondary care. iCARDIO 2025 recommends (R) remote HF monitoring devices to guide management and reduce hospitalisations [23].
Phase 3 - Optimise & Maintain (Week 12 onward)					
Phase 3 - Optimise & Maintain (Week 12 onward)	Comorbidity management: DM, HTN, sleep apnoea, AF, Valvular disease, and iron deficiency. Obesity management with GLP-1RA (semaglutide/tirzepatide) in HFpEF patients with obesity	-	-	-	Structured follow-up every 3-6 months. IV iron for iron deficiency anaemia, where available. Emphasise medication adherence and salt/fluid restriction. iCARDIO 2025 strongly recommends (SR) GLP-1RA (semaglutide, tirzepatide) in obese HFpEF patients to improve symptoms and QoL (STEP-HFpEF, SUMMIT trials). IV ferric carboxymaltose or ferric derisomaltose strongly recommended (SR) for iron deficiency to improve symptoms and reduce HF hospitalisations. Fluid restriction: iCARDIO 2025 recommends (R) restricting fluids to ~2 L/day based on FRESH-UP trial data [23].
Key Monitoring Parameters					
All phases	BP (target SBP ≥90 mmHg symptomatic threshold); HR; symptoms (dyspnoea, oedema); NYHA class	-	Serum creatinine & eGFR; serum K+; NT-proBNP (if available); body weight (daily)	-	Monitor at 1-2 weeks post-initiation, then at 4-6 weeks, then every 3 months. Simplified monitoring checklists recommended for non-specialist settings. iCARDIO 2025 supports simplified monitoring protocols appropriate for LMIC/resource-limited settings [23].

Bridging the evidence-practice gap

Delays in GDMT initiation have measurable consequences. Observational analyses indicate that, for every month of delay in achieving full GDMT, mortality risk rises substantially, as the greatest benefit is observed within the first 30-60 days of therapy initiation. Knowledge gaps regarding sequencing, tolerability thresholds, and laboratory monitoring lead to therapeutic hesitancy and prolonged exposure to uncontrolled disease [43,44]. Structured titration programs, incorporating nurse-led clinics and pharmacist support, have emerged as practical solutions. Such programs employ individualized schedules, simplified dose-escalation algorithms, and patient education modules to enhance adherence and minimize discontinuations [45]. Comparative studies across healthcare systems highlight the importance of multidisciplinary care in closing GDMT gaps [46]. Centres adopting integrated HF pathways with standardized discharge checklists and follow-up within seven days report markedly higher rates of quadruple therapy implementation and lower early readmissions. These successes highlight that the challenge is operational, not evidentiary: the efficacy of GDMT is unequivocal, but its delivery remains inconsistent [46].

Regional considerations and implementation challenges

In India and other emerging economies, HF patients are often younger, more frequently have ischemic or diabetic cardiomyopathy, and present at advanced stages. Economic constraints and fragmented insurance coverage limit consistent access to guideline-recommended drugs. In the Indian context, implementation of GDMT is influenced by cost constraints, limited access to newer therapies such as ARNIs and SGLT2is, and variability in healthcare infrastructure. Data from Indian registries and expert consensus statements highlight suboptimal uptake of GDMT and emphasize the need for region-specific strategies, including early combination therapy, patient education, and simplified treatment algorithms. Registries have documented underutilization of ARNIs and SGLT2is, and suboptimal titration of ACEis and BBs due to hypotension, renal impairment, or lack of monitoring. Addressing these gaps demands a system-wide strategy - incorporating cost-effective generic formulations, inclusion of key GDMT agents in national essential-medicine lists, and region-specific consensus statements to guide practice in resource-limited contexts. Rural and resource-limited settings can adhere to practical approaches, such as telemedicine-based dose-titration follow-up, technology-based task-sharing approaches that allow trained primary care physicians and community health workers to monitor dose-titration parameters, simpler monitoring algorithms (based on SBP, basic renal function), and community-based medication adherence programs. Equally crucial is clinician education: awareness of trial evidence, proper sequencing, and laboratory safety thresholds can mitigate reluctance to implement high-intensity regimens [3,5,6,13,21,24,47].

Future progress in HF management hinges less on discovering new drug classes and more on achieving universal, timely, and sustained application of existing GDMT. Integration of decision-support systems within electronic health records, outcome-based audits, and value-based reimbursement can incentivize adherence to guideline metrics. Translating clinical-trial efficacy into real-world effectiveness will require overcoming economic and infrastructural barriers while ensuring continuous follow-up for titration and monitoring [46,47].

Expert Opinion

GDMT remains the cornerstone of HFrEF management and should be initiated early - preferably simultaneously - and titrated effectively to reach maximum tolerated doses.

For most HFrEF patients, the initiation of all four pillars of GDMT within two weeks is recommended to achieve optimal clinical benefit. However, the sequencing and up-titration of these therapies should be individualized based on each patient's baseline hemodynamic status, renal function, and serum potassium levels to ensure safety and tolerability while maximizing therapeutic outcomes. Optimal clinical benefit can be achieved by initiation of all four pillars of GDMT within two weeks for most HFrEF patients based on expert consensus supported by the STRONG-HF trial [26]. The maximum tolerated doses are the highest recommended dose based on guidelines without symptoms of hypotension (SBP < 90 mmHg with symptoms), clinically significant renal deterioration (> 30% rise in serum creatinine from baseline), or persistent hyperkalemia (> 5.5 mEq/L). An alternative approach (consensus-based) could be sequential therapy of BBs and SGLT2is (not requiring titration), followed by ARNIs/ACEis and MRAs as hemodynamics and renal function permit in patients where SBP < 100 mmHg, significant renal impairment (eGFR < 30 mL/min/1.73m²), or baseline hyperkalemia when simultaneous initiation of therapy is not possible. It is recommended to conduct a clinical reassessment, which includes blood pressure, serum creatinine, potassium, and eGFR after one to two weeks of initiation of therapy and at each dose titration step, followed up every two to four weeks during the up-titration phase and every three to six months when stable doses are achieved (Table 2).

ARNI in GDMT: A game-changer

The introduction of ARNIs represents a transformative milestone in the treatment of HFrEF. This drug class combines two complementary mechanisms - neprilysin inhibition and angiotensin-II receptor blockade - to achieve broader neurohormonal modulation than previously possible with ACEis or ARBs alone. Neprilysin, a membrane-bound endopeptidase, catalyses the degradation of endogenous vasoactive peptides, such as atrial and B-type natriuretic peptides, bradykinin, and adrenomedullin. These peptides exert vasodilatory, natriuretic, diuretic, and anti-fibrotic effects that oppose the maladaptive activation of the RAAS and SNS. When neprilysin is inhibited, these protective pathways are amplified, improving hemodynamic balance and ventricular wall stress. However, neprilysin also degrades angiotensin II; thus, inhibiting it in isolation can paradoxically elevate circulating angiotensin II levels and offset benefit. Valsartan, an angiotensin II type-1 receptor blocker, prevents this counter-regulatory rise, ensuring sustained vasodilation and anti-remodelling effects. Early experimental studies demonstrated that dual RAAS and neprilysin inhibition improved cardiac output, reduced filling pressures, and reversed structural remodelling more effectively than single-pathway blockade; these observations were further confirmed in human hemodynamic studies. It is important to note that pivotal ARNI trials had under-representation of patients aged >75 years, severe renal impairment (eGFR < 20 mL/min/1.73m²) patients, New York Heart Association (NYHA) Class IV symptom patients, and certain ethnic populations, which limits the extrapolation of trial results to such populations. However, the first clinical attempts using omapatrilat (a combined ACE-neprilysin inhibitor) were halted due to excess bradykinin-mediated angioedema. Sacubitril/valsartan overcame this limitation by pairing neprilysin inhibition with ARB therapy, instead of ACE inhibition, providing potent efficacy with a safer bradykinin profile. This rational design laid the foundation for what has become one of the most practice-changing developments in HF pharmacotherapy [9].

Evidence from PARADIGM-HF and beyond

Primary Evidence: The PARADIGM-HF Trial

The pivotal PARADIGM-HF trial [9] enrolled 8,442 patients with symptomatic HFrEF (NYHA II-IV, EF ≤ 35%). Participants underwent sequential run-in phases with enalapril and then sacubitril/valsartan to ensure tolerability before randomization. Over a median follow-up of 27 months, sacubitril/valsartan achieved a 20% RRR in the composite endpoint of cardiovascular death or first HF hospitalization compared with enalapril, prompting early trial termination for overwhelming efficacy. It is worth noting that the sequential run-in design demonstrated tolerability to both enalapril and sacubitril/valsartan, thereby excluding 20% of screened patients, which might have overestimated tolerability and limited external validity when extrapolating these data to unselected population groups.

Key Clinical Outcomes From PARADIGM-HF

Breaking down the outcomes: Cardiovascular mortality was 20% lower (13.3% vs 16.5%), first HF hospitalization was reduced by 21% (12.8% vs 15.6%), and all-cause mortality declined by 16% (17.0% vs 19.8%). Improvements were consistent across age, sex, baseline ejection fraction, and geographic region. Patients also reported better quality-of-life scores on the Kansas City Cardiomyopathy Questionnaire (KCCQ), reflecting tangible symptom relief. Importantly, the incidence of renal impairment and hyperkalemia was comparable to enalapril, while cough was less frequent, confirming its favourable safety profile.

Supporting Trials: PIONEER-HF and TRANSITION

Subsequent trials reinforced these findings in acute and post-discharge settings. The results from the PIONEER-HF trial demonstrated that in-hospital introduction of sacubitine/valsartan in acute decompensated HFrEF patients showed a significant reduction in the surrogate biomarker NT-proBNP (the primary endpoint) at eight weeks compared to enalapril, with no additional adverse events. However, the trial did not show any differences in clinical outcomes such as mortality or rehospitalization. TRANSITION, a feasibility study, demonstrated that initiation of therapy before discharge or within 14 days thereafter was reasonable and well-tolerated, and the majority of patients could achieve the target dose in 10 weeks. This study's focus was on the proportion of people reaching the target dose rather than the clinical outcomes. These data collectively established that ARNI therapy can be started early, safely, and aggressively to maximize benefit [9].

Guideline Integration and Global Perspective

The consistency of benefit across diverse populations led to the swift and broad incorporation of ARNIs into international HF guidelines. The 2022 AHA/ACC/HFSA Heart Failure Guideline recommends sacubitril/valsartan as the first-line RAAS inhibitor in symptomatic HFrEF (Class I recommendation) [39], while the 2021 ESC Heart Failure Guideline [40] recommends switching from ACEi/ARB to ARNI (Class I recommendation) in symptomatic patients despite optimal treatment and a Class IIb recommendation for de novo initiation. There is quite an intriguing point of divergence in the guidelines on de novo ARNI initiation without prior ACEi/ARB exposure, where the ESC is more cautious than the AHA/ACC position. These frameworks focus on rapid sequencing as opposed to sequential replacement. The global recommendations were adapted to the Indian context by the Heart Failure Society of India (HFSI) 2022 consensus statement [21,24] by advising lower starting doses, extended titration intervals, and realistic monitoring schedules, which require limited access to specialists and reduced cost burden. Both frameworks emphasize rapid sequencing rather than sequential replacement, recognising that therapeutic delay perpetuates residual risk. In India, the HFSI 2022 Consensus Statement [21,24] mirrors these global recommendations while acknowledging real-world implementation barriers. These include drug-cost concerns, physician hesitation in initiating or titrating ARNI due to hypotension or renal considerations, and logistical challenges in ensuring frequent follow-up for dose escalation. The consensus highlights the importance of education, multidisciplinary HF clinics, and inclusion of ARNI in insurance or government-supported essential-medicine lists to enhance accessibility.

Mechanistic and Clinical Implications

The superiority of sacubitril/valsartan derives from its ability to concurrently suppress RAAS and amplify natriuretic-peptide signalling, restoring the physiological balance between vasoconstrictor and vasodilator forces. Neprilysin inhibition enhances cyclic-GMP-mediated pathways, improves arterial compliance, and attenuates myocardial fibrosis - mechanisms corroborated by imaging and biomarker studies demonstrating reverse remodelling, improved LVEF, and sustained NT-proBNP reductions. The drug's benefits extend beyond hemodynamics. Validated patient-reported outcome measures (i.e., KCCQ) demonstrated quality-of-life improvement in PARADIGM-HF and were associated with hard clinical endpoints. Renal protection signals are primarily from post-hoc analyses and secondary endpoints, revealing slower eGFR decline when compared to enalapril. However, none of the specific renal outcomes trials have verified these endpoints; thus, they must be viewed as being hypothesis-generating and not proven [9].

Pragmatic use of ARNI: Real-world applications

Despite the strong evidence from pivotal trials and consistent endorsement by major guidelines, real-world adoption of ARNIs remains suboptimal across both developed and emerging healthcare systems. Registry data consistently reveal that prescription and up-titration rates fall significantly short of expectations, with less than one-third of eligible patients receiving ARNI and an even smaller proportion achieving target doses [24]. This gap highlights the persistent disconnect between evidence-based recommendations and practical implementation.

In clinical practice, most physicians initiate therapy conservatively, often extending titration over six to eight weeks to ensure tolerability - particularly in patients with low baseline SBP (< 100 mmHg). The TITRATION trial [21] offered insight into this pragmatic approach, demonstrating that gradual dose escalation led to a higher rate of treatment success with fewer hypotensive events compared with rapid up-titration. Renal dysfunction thresholds that require dose adjustment are generally defined as a > 30% increase in serum creatinine or eGFR decline below 30 mL/min/1.73m². The correlation between sub-target dose and outcomes observed from real-world registries is merely observational and should not be interpreted as causal. However, these data suggest that even 50% of the target dose (sacubitril/valsartan: 49/51 mg BID) yields clinically significant improvements in hospitalization, but with reduced effect magnitude compared to full-target-dose therapy as demonstrated in PARADIGM-HF (Table 3).

**Table 3:** Practical guide to ARNI (sacubitril/valsartan) in heart failure: Eligibility, dose escalation, safety management, special populations, and structured follow-up with India-specific adaptations ARNI = angiotensin receptor-neprilysin inhibitor; ACEi = angiotensin-converting enzyme inhibitor; ARB = angiotensin receptor blocker; HFrEF = heart failure with reduced ejection fraction; HFpEF = heart failure with preserved ejection fraction; HFmrEF = heart failure with mildly reduced ejection fraction; EF = ejection fraction; SBP = systolic blood pressure; eGFR = estimated glomerular filtration rate; K+ = serum potassium; BD = twice daily; CKD = chronic kidney disease; DM = diabetes mellitus; CCB = calcium channel blocker; GWTG-HF = Get With the Guidelines-Heart Failure; HFSI = Heart Failure Society of India; NT-proBNP = N-terminal pro-B-type natriuretic peptide; NNT = number needed to treat; NYHA = New York Heart Association; IHD = ischaemic heart disease; RR = rate ratio; CI = confidence interval; SZC = sodium zirconium cyclosilicate; BMI = body mass index; TTR = transthyretin; MDT = multidisciplinary team; GLP-1RA = glucagon-like peptide-1 receptor agonist; SR = strongly recommend; R = recommend

Parameter	Clinical Scenario/Threshold	Recommended Action	India-Specific Consideration
Eligibility & Prerequisites			
Indication	HFrEF (EF ≤40%) NYHA II-IV; symptomatic on optimal therapy; selected HFmrEF/HFpEF (EF 41-57%), especially women	Preferred over ACEi/ARB in all eligible HFrEF patients. Consider HFpEF where EF ≤57% and in women	Indian guideline (HFSI 2022) endorses ARNI as a first-line RAAS inhibitor. Limited awareness among general physicians; specialist initiation is often required. iCARDIO 2025 recommends (R) ARNI in all HFrEF patients and in HFpEF. Resource-limited settings may use ACEi/ARB as an alternative per iCARDIO economic adjustment guidance [23].
Prerequisite	Washout period of 36 hours required if switching from ACEi (to avoid angioedema); not required when switching from ARB	Hold ACEi for ≥36 hours before first sacubitril/valsartan dose. Direct switch from ARB is safe	Many Indian patients are on enalapril/ramipril - educate treating physicians on the mandatory washout period to prevent angioedema.
Contraindications	SBP <90 mmHg (symptomatic); history of angioedema with ACEi/ARB; eGFR <30 mL/min (relative); K+ >5.4 mEq/L; pregnancy; concomitant ACEi use	Do not initiate. Address the underlying cause (e.g., optimise diuretics, correct electrolytes) and reassess	Renal dysfunction is common in Indian HF patients (often coexisting CKD/DM). Reassess eligibility after optimising fluid status and nephrotoxic drugs.
Initiation Doses			
SBP ≥100 mmHg (haemodynamically stable; any prior RAAS therapy)	Haemodynamically stable; on optimal background therapy	Start: Sacubitril/valsartan 49/51 mg BD (target: 97/103 mg BD)	Feasible for the majority of stable outpatients. Confirm renal function and K+ prior to initiation.
SBP 90-99 mmHg or Frail/Elderly	Low but acceptable BP; patient on multiple antihypertensives or diuretics	Start: Sacubitril/valsartan 24/26 mg BD; review contributing BP-lowering agents (reduce/stop CCBs, nitrates, excess diuretics)	Very common scenario in Indian practice. Many patients are on amlodipine/nitrates for IHD. Down-titrate these before ARNI, not the ARNI itself.
In-hospital Initiation (acute HF, stabilised)	PIONEER-HF/TRANSITION evidence: Safe if haemodynamically stable prior to discharge (SBP ≥100 mmHg for ≥6 hrs, no IV diuretics/vasodilators)	Start: 24/26 mg or 49/51 mg BD before or within 14 days of discharge → Superior NT-proBNP reduction vs. enalapril at 8 weeks	Inpatient initiation is feasible but requires physician familiarity. Structured discharge checklists help. Use a lower starting dose if the SBP is 90-100 mmHg at discharge.
Up-Titration Schedule			
Step 1	Weeks 0-2: Starting dose 24/26 mg BD or 49/51 mg BD	Check BP, renal function, K+ at 1-2 weeks; If tolerated → escalate	The first review at 1-2 weeks is critical. Teleconsultation is acceptable where an in-person visit is not feasible.
Step 2	Weeks 2-4: Intermediate dose 49/51 mg BD (if started at low dose)	Double dose if SBP ≥100 mmHg and K+ <5.0, eGFR stable; continue if asymptomatic even at SBP 90-99 mmHg	Many Indian patients plateau here due to low BP. Maintaining 49/51 mg BD still confers significant clinical benefit over ACEi/ARB.
Step 3	Weeks 4-8: Target dose 97/103 mg BD	Escalate to target if tolerated; If not tolerated, maintain the highest tolerated dose - do not discontinue	The target dose may not be achievable in all patients. Sub-target doses (49/51 mg BD) still demonstrate meaningful HF hospitalization reduction in Indian real-world data.
Slow Titration (if needed)	TITRATION trial: Gradual escalation over 6-8 weeks preferred in patients with SBP concerns. Retrospective multicentre cohort, n=1,039+ Indian HFrEF patients, LVEF ≤40%, NYHA II-IV; 160 centres pan-India): Largest Indian real-world study specifically designed to evaluate hypotension incidence and optimal dosing/titration strategies for sacubitril/valsartan in HFrEF - directly validates the need for structured dose escalation protocols (50 mg → 100 mg → 200 mg BD) in Indian clinical practice; results anticipated to provide India-specific benchmarks for hypotension rates and dose down-titration frequency	Extend titration interval to 4-6 weeks per step if BP is borderline or patient is elderly/frail; prioritise persistence over speed	Slow titration is the pragmatic approach for most Indian patients. Acceptable and recommended by the HFSI consensus.
Managing Common Adverse Effects			
Symptomatic Hypotension (SBP <90 mmHg with symptoms)	Dizziness, presyncope, and fatigue associated with low BP readings	(1) Reduce/stop diuretics, CCBs, nitrates. (2) Down-titrate ARNI one step. (3) Discontinue only if refractory to the above measures	Nitrates for IHD and diuretics for congestion are common contributors. Address these first before reducing ARNI.
Asymptomatic Low SBP (SBP 90-99 mmHg, no symptoms)	BP low on reading but the patient feels well; no dizziness, no signs of hypoperfusion	Continue ARNI at current dose with close monitoring; do NOT reduce or stop for asymptomatic low readings alone	Physician hesitancy at low SBP is a key barrier in India. Asymptomatic low SBP should not trigger dose reduction if the patient is clinically stable.
Renal Dysfunction (Rise in creatinine)	Creatinine rise ≤30% from baseline or eGFR decline to 25-30 mL/min	Reduce diuretic dose; recheck in 1-2 weeks; mild transient rise is expected and acceptable; discontinue only if eGFR <20 or rapid deterioration	Coexisting DM and CKD are prevalent in Indian HF. Mild creatinine rises are common and usually reversible. Avoid premature ARNI discontinuation.
Hyperkalaemia (K+ >5.0 mEq/L)	K+ 5.0-5.4: Caution; K+ >5.4: Hold/reduce dose	Reduce/stop MRA first; optimise dietary K+ intake; consider K+-binders if available; recheck electrolytes in 1 week	Potassium-binders (patiromer, SZC) have limited availability in India. Dietary counselling on K+ restriction is an important low-cost adjunct.
Angioedema	Rare; more common if switched from ACEi without adequate washout	Discontinue immediately if angioedema occurs; ensure a 36-hour ACEi washout is observed	Educate patients and families on angioedema recognition. Ensure washout period is strictly followed - key gap in awareness among Indian prescribers.
Special Populations			
Women with HFpEF/HFmrEF (EF ≤57%)	PARAGON-HF: 27% reduction in HF hospitalisations in women (RR 0.73; 95% CI: 0.59-0.90); Pooled PARAGLIDE + PARAGON data support benefit. Per Foà et al. [48]: benefit/risk ratio favours sacubitril/valsartan in LVEF <60% (prevents ~3 primary events per 1 extra hypotensive episode); LVEF ≥60% associated with higher hypotension risk and no primary event reduction - reinforce LVEF <60% as key threshold. Kommu et al. [30] (4 RCTs, n=6,737): pooled composite HF hosp + CV death RR 0.86 (95% CI: 0.75-0.99, p=0.04); KCCQ-CSS MD +1.13 (p=0.024); NYHA class improvement OR 1.32 (p=0.002); no significant CV death or all-cause mortality benefit; hypotension OR 1.67 (95% CI: 1.27-2.19, p<0.0001) but no excess hyperkalaemia (OR 0.90) or worsening renal function (OR 0.80)	Consider ARNI in addition to an SGLT2 inhibitor in women with EF ≤57% and patients with LVEF <60%; start at low dose; titrate cautiously. Per Foà et al. [48], exercise heightened vigilance for hypotension in patients with LVEF ≥60%, where benefit is not demonstrated. Kommu et al. [30] confirmed hypotension as the predominant safety concern with ARNI in HFpEF/HFmrEF (OR 1.67 vs valsartan), while renal function and potassium levels are not disproportionately worsened - relevant for Indian patients with coexisting CKD and T2DM	HFpEF is underdiagnosed in Indian women. The ARNI-PRESERVED Indian study supports feasibility. Emphasis on echocardiographic follow-up needed. iCARDIO 2025 recommends (R) ARNI in HFpEF patients; recommends (R) SGLT2i (SR) + MRA (R) as foundational HFpEF therapy with GLP-1RA (SR) in obese HFpEF - directly relevant to Indian women with HFpEF [23].
Elderly/Frail Patients	Age >70, low BMI, multiple comorbidities, polypharmacy	Begin at 24/26 mg BD; extend titration intervals (every 4-6 weeks); Prioritise tolerability and persistence	Indian HF patients often present at younger ages but with advanced disease. Frailty assessment and simplified drug regimens improve adherence.
CKD (eGFR 30-60 mL/min)	Mild-to-moderate CKD coexisting with HF	ARNI can be initiated; start low; Monitor creatinine and K+ closely at 1-2 weeks; avoid if eGFR <30 mL/min	CKD is highly prevalent in Indian HF (co-occurring DM and hypertension). Renal monitoring infrastructure must be in place before initiation.
TTR Amyloid Cardiomyopathy	HF patients with suspected/confirmed TTR amyloidosis (wild-type or hereditary)	Screen with serum/urine immunofixation + serum free light chains (SR) and DPD/PYP/HMDP scintigraphy (SR). Initiate tafamidis, acoramidis, or vutrisiran if confirmed (SR per iCARDIO 2025). Avoid standard GDMT drugs (ACEi, ARB, ARNI, MRA, beta-blockers) that may be poorly tolerated in infiltrative cardiomyopathy	iCARDIO 2025 strongly recommends (SR) tafamidis/acoramidis/vutrisiran for TTR amyloid cardiomyopathy (ATTRibute-CM and HELIOS-B trial evidence). In resource-limited settings, diflunisal may be considered (R). Refer to MDT/Neurology (R). Increasing recognition in India; genetic testing (SR) important for hereditary forms [23].
Structured Follow-Up Schedule			
Weeks 1-2	First post-initiation review	Check BP, HR, weight, symptoms, renal function, K+; assess tolerability; escalate if stable	Teleconsultation or phone follow-up is acceptable if an in-person visit is not feasible. Nurse-led HF clinics can conduct structured monitoring.
Weeks 4-6	Second titration review	Assess for dose escalation to target; Repeat labs; adjust diuretics as needed; NT-proBNP measurement if available	NT-proBNP monitoring is not universally available; clinical assessment of symptoms and functional status is an acceptable surrogate.
3 Monthly (maintenance)	Long-term monitoring	GDMT optimisation review, NYHA class, adherence, BP/HR, echocardiography annually or as indicated	Annual echocardiography may not be feasible in all settings. Focus on clinical assessment and GDMT adherence reviews. National HF registry enrolment encouraged. iCARDIO 2025 supports non-invasive home tele-monitoring (Su) as an adjunct to structured care in resource-limited settings [23].

These findings have informed current prescribing behaviour, with many clinicians prioritizing dose persistence and patient stability over rapid attainment of target doses. Importantly, data from real-world registries show that, even sub-target doses confer significant reductions in hospitalizations and cardiovascular events, emphasizing that maintaining therapy - rather than reaching a specific numeric goal - is often the more meaningful determinant of long-term outcomes.

However, several barriers continue to hinder optimal use. Symptomatic hypotension remains the most common reason for either withholding or discontinuing therapy, particularly in elderly or frail patients. In one single-centre study, more than 85% of patients who failed to reach target doses reported low blood pressure as the primary limiting factor, while renal dysfunction was the second most frequent contributor [21]. Although mild renal function changes are common after initiation, clinically significant impairment is rare and usually reversible with dose adjustments. The optimal use of ARNI is affected by major barriers at various levels: (a) systemic barriers include disintegrated healthcare delivery, lack of structured pathways after discharge, lack of insurance coverage, ineffective government health schemes, and poor integration of ARNI into essential medicine lists; (b) economic barriers include high medication costs, regional differences in drug prices and generic availability of drugs, and competing financial priorities within the household; and (c) physician-level barriers include therapeutic inertia, limited knowledge of titration regimens and drug safety monitoring, managing hypotension without specialist support, and suboptimal awareness of evolving guideline recommendations (Table 4). These multilevel issues contribute to the underutilization of ARNI [48]. Addressing these limitations requires a practical, individualized approach to initiation and titration. Starting therapy at a very low dose in patients with borderline SBP, extending the up-titration interval, and closely monitoring renal function and electrolytes have all proven effective in improving tolerability. Adjusting diuretics or other blood pressure-lowering drugs, such as calcium channel blockers or nitrates, before ARNI initiation can mitigate early hypotensive episodes without compromising long-term benefit. Structured patient education - reinforcing adherence, expected transient symptoms, and the importance of regular follow-up - has emerged as a crucial component of success. Incorporating nurse-led HF clinics, pharmacist follow-up, or teleconsultation has been shown to enhance adherence and dose escalation, especially in busy or resource-limited settings [49].

**Table 4:** Actionable recommendations for improving GDMT and ARNI uptake in India ACEi = angiotensin-converting enzyme inhibitor; ARB = angiotensin receptor blocker; ARNI = angiotensin receptor-neprilysin inhibitor; ARR = absolute risk reduction; BD = twice daily; BP = blood pressure; CV = cardiovascular; DKA = diabetic ketoacidosis; DM = diabetes mellitus; eGFR = estimated glomerular filtration rate; GLP-1RA = glucagon-like peptide-1 receptor agonist; GWTG-HF = Get With the Guidelines-Heart Failure; HF = heart failure; HFmrEF = heart failure with mildly reduced ejection fraction; HFpEF = heart failure with preserved ejection fraction; HFrEF = heart failure with reduced ejection fraction; HR = hazard ratio; K+ = serum potassium; KCCQ-CSS = Kansas City Cardiomyopathy Questionnaire Clinical Summary Score; LMIC = low- and middle-income countries; LVEF = left ventricular ejection fraction; MRA = mineralocorticoid receptor antagonist; NT-proBNP = N-terminal pro-B-type natriuretic peptide; NNT = number needed to treat; NYHA = New York Heart Association; QoL = quality of life; RR = rate ratio; RRR = relative risk reduction; SBP = systolic blood pressure; SGLT2i = sodium-glucose cotransporter-2 inhibitor; SR = strongly recommend; T2DM = type 2 diabetes mellitus; TTR = transthyretin; 6MWT = 6-minute walk test

Recommendation	Evidence Level	Target Stakeholder	Expected Impact
Initiate all four GDMT pillars (ARNI/ACEi/ARB, beta-blocker, MRA, SGLT2i) simultaneously or within two weeks of diagnosis/hospital admission; sequencing should be individualized based on hemodynamic status, renal function, and serum potassium.	Trial-supported: STRONG-HF [26]; DAPA-HF [8]; EMPEROR-Reduced [24]; PARADIGM-HF [9]; Packer et al. [24]. Registry-supported: GWTG-HF registry [27]; n=33,036 newly diagnosed HFrEF): >80% eligible for quadruple GDMT; only 15% prescribed quadruple GDMT and 42% triple therapy in contemporary practice; in-hospital quadruple GDMT initiation yields up to 25% absolute risk reduction in 1-year mortality (NNT=4). Rapid/simultaneous initiation advocates starting at least low doses of all 4 medications simultaneously or within 1 week as an outpatient, or by the time of discharge if hospitalised [25]	Clinician	Reduced 180-day all-cause mortality and HF readmissions; improved neurohormonal modulation across all four therapeutic axes [26,36]. GWTG-HF registry [27]: in-hospital quadruple GDMT initiation yields up to 25% absolute risk reduction in 1-year mortality (NNT=4). In-hospital initiation of BB, ARNI, and MRA associated with a > 3-fold increase in post-discharge medication adherence vs deferred initiation.
Prefer sacubitril/valsartan (ARNI) over ACEi/ARB as the first-line RAAS inhibitor in symptomatic HFrEF when hemodynamically stable.	Trial-supported: PARADIGM-HF [9]; AHA/ACC/HFSA and ESC guidelines [39,40]	Clinician	20% relative risk reduction in CV death/HF hospitalization; 16% lower all-cause mortality vs enalapril [9]
Initiate ARNI in-hospital (during admission for acute decompensated HF) once the patient is hemodynamically stable; therapy may also be started before discharge or within 14 days thereafter.	Trial-supported: PIONEER-HF and TRANSITION [9]	Clinician	Greater NT-proBNP reduction at 8 weeks; feasibility of pre-discharge initiation confirmed [9]
In patients with low or borderline systolic BP, start ARNI at a very low dose with gradual up-titration to maximum tolerated dose; if asymptomatic low BP develops, continue ARNI with close monitoring; if symptomatic hypotension occurs, evaluate contributing factors and consider down-titrating or discontinuing diuretics, calcium channel blockers, or nitrates.	Consensus-based: TITRATION trial [21]; HFSI consensus [21,47]; Indian expert opinion [21,47]. Jadhav et al. [50]; n=1,039+ Indian HFrEF patients, LVEF ≤40%, NYHA II-IV; 160 centres pan-India): Largest ongoing Indian real-world study evaluating hypotension incidence and dose titration strategies for sacubitril/valsartan in HFrEF - specifically designed to quantify down-titration rates and reasons for treatment discontinuation in Indian clinical practice; directly supports this recommendation	Clinician	Improved tolerability and therapy persistence; reduction in symptomatic hypotension without compromising long-term benefit [21]. Jadhav et al. [50] will prospectively quantify hypotension incidence, up-titration time to 200 mg BD, and down-titration rates across 160 Indian centres - results anticipated to provide the first large-scale India-specific benchmarks for hypotension-driven dose adjustment and treatment discontinuation rates in real-world HFrEF practice
Monitor renal function and electrolytes at initiation and after each dose up-titration; mild renal function changes are common after initiation - clinically significant impairment is rare and usually reversible with dose adjustment; do not discontinue ARNI prematurely for mild, asymptomatic changes.	Consensus-based: HFSI consensus [21,47]; Indian expert opinion [21,47]	Clinician	Maintained patient safety while preserving long-term benefit; reduces unnecessary therapy discontinuation [21,47]
Consider ARNI in women with HFpEF and in patients with HFmrEF (LVEF 40-49%, per ESC 2021 definition) as an adjunct to SGLT2 inhibitors to reduce HF hospitalization risk. The benefit appears greatest in patients with LVEF up to 57% and in women. Foà et al. [48] further showed that the benefit-risk profile of sacubitril/valsartan is most favourable when LVEF is below normal (<60%): in patients with LVEF <60%, approximately three primary events are prevented for every one additional hypotensive episode, whereas in those with LVEF ≥60%, no primary events are prevented and about three additional hypotensive episodes occur per 100 patients. Accordingly, clinicians should prioritize sacubitril/valsartan in HFmrEF/HFpEF patients with LVEF <60% and exercise greater caution with hypotension monitoring in those with LVEF ≥60%. In addition, Kommu et al. [49], in a meta-analysis of four RCTs (n=6,737), reported that sacubitril/valsartan reduced the composite of HF hospitalization and cardiovascular death (RR 0.86, 95% CI: 0.75-0.99; p=0.04), improved KCCQ-CSS (MD +1.13; p=0.024), and improved NYHA class (OR 1.32; p=0.002), but did not significantly reduce cardiovascular or all-cause mortality. Hypotension was more frequent (OR 1.67, 95% CI: 1.27-2.19; p<0.0001), whereas no significant excess in hyperkalaemia (OR 0.90) or worsening renal function (OR 0.80) was observed, indicating that hypotension monitoring, rather than renal or electrolyte concerns, should remain the primary safety focus in clinical practice [30].	Trial-supported: PARAGON-HF [29] (27% reduction in HF hospitalization in women); PARAGLIDE-HF pooled analysis [28,32]; 2024 ACC Expert Consensus [51]; ESC 2021 [39]; Foà et al. [48] (LVEF <60% as benefit/risk threshold for sacubitril/valsartan in HFpEF/HFmrEF); Kommu et al. [49] (pooled RCT meta-analysis confirming composite HF hosp + CV death RR 0.86, hypotension as primary safety concern, no excess renal or electrolyte risk)	Clinician	Reduced HF hospitalizations; improved NYHA functional class and KCCQ quality-of-life scores [29,32]. Foà et al. [48] confirms in LVEF <60%, sacubitril/valsartan prevents ~3 primary events (CV death/HF hospitalisation) per 1 extra hypotensive episode vs valsartan; in LVEF ≥60%, the risk/benefit balance does not favour sacubitril/valsartan. Post-hypotension prognosis is adverse (adjusted RR 1.63, p<0.001 for primary endpoint; HR 1.62 for all-cause mortality), but sacubitril/valsartan treatment benefit remains consistent irrespective of hypotension occurrence. Kommu et al. [49] further confirms: composite HF hosp + CV death RR 0.86 (95% CI: 0.75-0.99); KCCQ-CSS MD +1.13; NYHA improvement OR 1.32; and importantly, no significant increase in hyperkalaemia (OR 0.90) or worsening renal function (OR 0.80) - providing reassurance for Indian patients with CKD and T2DM who may otherwise be considered higher risk for ARNI initiation
Initiate GLP-1 receptor agonist (semaglutide or tirzepatide) in patients with HFpEF and obesity (BMI ≥30 kg/m²) to improve symptoms, exercise capacity, and quality of life, and to reduce worsening HF events; combine with SGLT2 inhibitor and MRA as foundational HFpEF therapy.	Trial-supported: STEP-HFpEF (Kosiborod et al., 2023); STEP-HFpEF DM (Kosiborod et al., 2024); pooled STEP-HFpEF analysis (Butler et al., 2024); SUMMIT - Tirzepatide (Packer et al., 2005); iCARDIO Alliance 2025 Guidelines [23] - strongly recommends (SR) GLP-1RA in obese HFpEF	Clinician	Semaglutide: ~7-point improvement in KCCQ-CSS and ~20-metre improvement in 6MWT vs placebo; tirzepatide (SUMMIT): 38% reduction in composite of worsening HF events or CV death (HR 0.62, 95% CI: 0.41-0.95); ~15% weight reduction. iCARDIO 2025 strongly recommends (SR) GLP-1RA for obese HFpEF patients to improve symptoms and QoL. Availability and cost remain barriers in India; advocate for formulary inclusion.
Consider finerenone as a non-steroidal MRA option in patients with HFpEF or HFmrEF (LVEF ≥40%), particularly those with coexisting T2DM and CKD, in addition to SGLT2 inhibitors; where finerenone is unavailable due to resource constraints, spironolactone (Super iCARDIO 2025) is an alternative.	Trial-supported: FINEARTS-HF (Solomon et al., 2024); iCARDIO Alliance 2025 Guidelines [23] - recommends (R) finerenone in HFpEF	Clinician	~16% reduction in CV death or worsening HF events (HR 0.84, 95% CI: 0.74-0.95); lower hyperkalaemia and gynaecomastia risk vs spironolactone. Per iCARDIO 2025 [23], finerenone is graded (R) above spironolactone (Su) for HFpEF. Particularly relevant for Indian HFpEF patients with T2DM and CKD. Where finerenone is unavailable, spironolactone (Su) is the accessible alternative per iCARDIO 2025 economic adjustment guidance
Align clinical practice with the iCARDIO Alliance Global Implementation Guidelines on Heart Failure 2025, which provide evidence-based recommendations explicitly adapted for resource-limited settings (including India/LMIC), with 'economic adjustment' guidance for situations where resources are 'somewhat limited' or 'severely limited.'	Guideline-supported: iCARDIO Alliance 2025 Guidelines [23]; incorporates FINEARTS-HF, STEP-HFpEF, SUMMIT, FAIR-HF2, ATTRibute-CM, HELIOS-B, RESHAPE-HF2, TRILUMINATE, MONITOR-HF, FRESH-UP trial	Clinician/Policymaker/Healthcare system	Provides universal, resource-stratified recommendations applicable to India and other LMIC; addresses barriers at health system, provider, and patient levels; explicitly recommends economic adjustment strategies to optimise HF care within resource constraints; enables region-specific guideline implementation
Implement structured post-discharge follow-up within 7 days (nurse-led or pharmacist-supported HF clinic/teleconsultation) with a standardised discharge checklist confirming all four GDMT components prescribed.	Trial-supported: STRONG-HF [26]; consensus-based: multidisciplinary HF pathway evidence [43,46]	Clinician/Healthcare system	Higher rates of quadruple therapy at discharge; lower early readmission rates [26,46]
Establish dedicated multidisciplinary HF clinics with structured titration protocols, individualized dose-escalation algorithms, and patient education modules.	Consensus-based: HFSI consensus [21,47]; international HF pathway evidence [43,46]	Healthcare system	Improved GDMT adherence; reduced therapeutic inertia; enhanced long-term patient outcomes [43,46]
Incorporate ARNI, SGLT2 inhibitors, and other guideline-recommended GDMT into national essential medicine lists and government/insurance reimbursement schemes.	Consensus-based: HFSI consensus [21,47]; Indian expert opinion [21,47]	Policymaker	Improved affordability and accessibility; reduced out-of-pocket cost burden for patients in resource-limited settings [21,47]
Develop and maintain a national heart failure registry to generate India-specific real-world data on GDMT utilisation, barriers, outcomes, and cost-effectiveness.	Consensus-based: HFSI consensus [21,47]; Indian expert opinion [21,47]	Policymaker/Healthcare system	Informs region-specific guidelines and treatment algorithms; identifies gaps in HFmrEF/HFpEF management [21,47]
Provide ongoing physician training on GDMT evidence, tolerability management, and rapid up-titration strategies, with particular focus on primary and secondary care centres.	Consensus-based: CHAMP-HF registry [11]; QUALIFY global survey [52]; Indian expert opinion [21,47]	Policymaker/Healthcare system	Reduction in physician hesitancy; improved prescribing rates and ARNI uptake beyond tertiary centres [11,52]
Integrate clinical decision-support tools within electronic health records to prompt GDMT initiation, flag eligible patients for ARNI, and alert for required monitoring.	Consensus-based: Zhang et al. [33]; Jain et al. [35]; Shahid et al. [52] - Trial-supported EHR strategies: BETTER-CARE trial: 52% vs 30% GDMT prescription improvement; EPIC-HF RCT: 49.0% vs 29.7% GDMT intensification at 30 days (p<0.001); POMPAF trial: 11% adjusted increase in beta-blocker prescriptions; MPEMF trial: improved ACEi/ARB and MRA rates; REVAL-HF: 47% vs 25% GDMT optimisation score increase	Healthcare system	Systematic reduction of missed opportunities for GDMT; supports adherence to guideline metrics [46,47]; EHR-integrated alerts and patient activation tools reduce clinical inertia: BETTER-CARE trial improved GDMT uptake by 22 percentage points; EPIC-HF patient activation tool achieved 19.3 percentage-point greater GDMT intensification at 30 days (P<0.001); combined EHR + multidisciplinary strategies are most effective [53]
Implement multicomponent, low-cost GDMT adherence interventions combining culturally tailored patient education, simplified once-daily/fixed-dose combination regimens, digital reminders (SMS), and community health worker-led follow-up; address socioeconomic barriers (medication cost, transportation) through task-sharing models and primary health system integration.	Prospective, multicenter real-world cohort (Herrmann et al. [54]; n=7,532 HFrEF patients, LVEF ≤40%, 15 Indian cities, January 2020-January 2024; 75 physicians): baseline GDMT compliance 32.3% (only 17.5% on ≥3 GDMT agents); post-intervention compliance 68.4% (p<0.001); 54.6% achieving ≥3 GDMT agents; key barriers - medication cost (OR 3.2, p=0.01), lack of transportation (OR 2.8, p=0.03), fear of side effects (OR 2.1, p=0.04); ARNI and CRT-D critically underutilised	Clinician/Policymaker/Healthcare system	GDMT compliance more than doubled (32.3% → 68.4%; p<0.001); proportion on ≥3 GDMT agents improved from 17.5% to 54.6%; hospitalizations for acute decompensated HF decreased 41.6% (p=0.002) in compliant patients; confirms that systematic, low-cost, multicomponent interventions significantly improve real-world GDMT adherence in resource-constrained Indian settings; provides the largest prospective Indian-specific evidence base (n=7,532, 15 cities) for interventions directly targeting the barriers (cost, transportation, health literacy, side-effect fear) identified in this manuscript; ARNI and CRT-D underutilisation identified as critical gaps requiring urgent policy action
Adopt “time-to-quadruple therapy” as a key quality performance measure for heart failure centres; track proportion of eligible HFrEF patients who receive all four GDMT pillars by time of hospital discharge or within 1-4 weeks of new HF diagnosis.	Quality metric framework: Shahid et al. [52] (cited in [25]). Time to quadruple guideline-directed medical therapy as a key performance measure for heart failure. Real-world context: GWTG-HF registry (Greene et al. [53]; n=33,036): only 15% of eligible HFrEF receive quadruple GDMT; 42% receive triple therapy; >80% are eligible but untreated to guideline-recommended quadruple regimen.	Policymaker/Healthcare system/Clinician	Promotes institutional accountability for timely GDMT initiation; creates measurable targets to combat clinical inertia; enables benchmarking of Indian HF centres against international standards; directly addresses the gap between eligibility (>80%) and actual prescription (15%) of quadruple GDMT in real-world practice; promotes culture shift toward “therapeutic urgency” endorsed by Shahid et al. [52]

In India and other low- and middle-income regions, additional socioeconomic factors play a role. Out-of-pocket expenses, limited access to specialist care, and inadequate systems for post-discharge monitoring reduce the feasibility of early and aggressive ARNI titration. Recognizing these constraints, the HFSI has encouraged a stepwise strategy emphasizing early inpatient initiation, low starting doses, and multidisciplinary management to optimize tolerability and persistence [21,49]. Real-world data from Indian cohorts demonstrate improved outcomes even at half-target doses, supporting the principle that maintaining patients on a tolerated regimen - rather than striving exclusively for full-dose therapy - is clinically meaningful in the local context.

Overall, the pragmatic use of ARNI hinges on early initiation, slow but steady up-titration, and sustained adherence, adapted to each patient's hemodynamic profile and clinical stability. When approached systematically, most patients can successfully tolerate therapy and experience measurable improvements in symptom burden and hospitalization risk (Table 3).

Expert Opinion

In HFrEF patients, ARNI should be initiated as early as possible over ACEis even if the patient is hemodynamically stable (SBP ≥ 100 mmHg) based on the randomized trial evidence, such as PARADIGM-HF, PIONEER-HF, and TRANSITION [9]. It is recommended to start with sacubitril/valsartan 24/26 mg BID and then increase gradually to the maximum tolerated dose (consensus-based) in patients with low SBP (90-100 mmHg). Assessment of blood pressure, serum creatinine, potassium, and eGFR is required at one to two weeks, with each dose titration step and continuous monitoring every one to three months until a stable dose is achieved. If asymptomatic low SBP develops after initiation, continuation of ARNI with close clinical monitoring is recommended. In cases of symptomatic hypotension, it is essential to evaluate and address contributing factors and consider down-titrating or discontinuing other blood pressure-lowering agents, such as diuretics, calcium channel blockers, or nitrates, where appropriate (see Table 3 for a practical ARNI initiation algorithm, including SBP-stratified dosing and adverse effect management).

ARNI in HFpEF: Expanding the therapeutic window

Therapeutic progress in HFpEF has historically lagged behind that of HFrEF, largely because of its heterogeneous pathophysiology and the absence of consistent mortality benefits from conventional neurohormonal antagonists. For decades, management has been centred on symptom relief with diuretics and control of comorbidities, such as hypertension, obesity, atrial fibrillation, and diabetes. Early trials with RAAS blockers and BBs showed only modest or neutral results, reflecting the complex interplay of vascular stiffness, systemic inflammation, and impaired ventricular-vascular coupling that typifies HFpEF.

The emergence of targeted therapies has gradually reshaped this landscape. SGLT2is have demonstrated reproducible, though modest, reductions in hospitalization risk and improvements in patient-reported outcomes. Against this evolving background, the introduction of ARNI marked a major conceptual advance by addressing both maladaptive neurohormonal activation and impaired natriuretic peptide signalling-pathways central to the HFpEF phenotype [32].

Primary Evidence: The PARAGON-HF Trial

The PARAGON-HF trial was the first large-scale randomized study to evaluate sacubitril/valsartan in HFpEF, enrolling 4,822 patients with an LVEF ≥45%. Although the trial narrowly missed its primary endpoint (rate ratio (RR): 0.87; 95% CI: 0.75-1.01; p = 0.06), it demonstrated clear signals of benefit in specific subgroups, particularly among women and those at the lower end of the preserved EF spectrum (≤ 57%) [28,29]. The gender-stratified analysis showed a significant 27% reduction in HF hospitalizations in women (RR: 0.73; 95% CI: 0.59-0.90), contrasting with a neutral effect in men (RR: 1.03; 95% CI: 0.84-1.25; p = 0.017) [55]. These data suggest sex-specific differences in myocardial and vascular responsiveness to neprilysin inhibition, possibly related to hormonal influences on natriuretic peptide activity and extracellular-matrix regulation. Beyond hospitalization rates, secondary analyses revealed consistent improvements in NYHA functional class, quality-of-life scores (KCCQ), and NT-proBNP levels, indicating better symptom control and hemodynamic stabilization even without a mortality signal. The parallel PARAGLIDE-HF and pooled meta-analyses combining PARAGON-HF with PARADIGM-HF further strengthened these observations, showing reduced composite rates of cardiovascular death or HF hospitalization and improved functional status among patients with EF values at the lower end of the "preserved" spectrum. These outcomes support the concept of a continuum of therapeutic responsiveness across EF categories; however, it is imperative to determine if these benefits have led to decreased HF hospitalization, as there is no statistically significant reduction in mortality in both HFpEF (LVEF ≥ 50%) or HFmrEF (LVEF 41-49%) populations. The HFmrEF subgroup was reported to have a consistent benefit over the HFpEF population. Moreover, the subgroup analyses that are stratified by EF range and sex are post-hoc and have intrinsic statistical constraints, such as multiplicity and the possibility of type 1 error. Therefore, these findings have to be considered as hypothesis-generating rather than conclusive evidence [29,32].

Real-World Evidence in HFpEF

In a real-world context, smaller observational studies have mirrored these findings. Jariwala et al. (ARNI-PRESERVED study) reported meaningful clinical improvement over six months, including upward shifts in NYHA class and improved exercise tolerance in Indian HFpEF patients treated with sacubitril/valsartan. This is one of the few Indian real-world studies that evaluated ARNI in HFpEF [43]. However, the findings from this study cannot be considered definitive evidence owing to the small sample size and single-centre design. Therefore, large, multicentre, prospective studies targeting Indian HFpEF and HFmrEF populations are required to validate these findings and refine patient selection criteria for the local context [33].

Mechanistic Basis for ARNI in HFpEF

Mechanistically, neprilysin inhibition in HFpEF enhances cyclic GMP signalling, mitigates arterial and ventricular stiffness, and improves ventricular-vascular coupling - key determinants of effort intolerance and elevated filling pressures. By countering endothelial dysfunction and promoting natriuresis, sacubitril/valsartan may also modulate systemic inflammation and microvascular rarefaction, which are now recognized contributors to the HFpEF syndrome. This physiological rationale aligns with the subgroup benefits observed in patients with obesity, diabetes, and hypertension - conditions characterized by heightened neurohormonal and oxidative stress. Pooled analyses of PARAGON-HF and PARADIGM-HF suggest that these phenotypes derive particular advantage from ARNI therapy [28,56]. Despite these promising signals, several practical barriers remain. Hypotension and renal function fluctuations are the most frequently cited concerns in real-world use. These effects, however, are generally mild and manageable with careful titration, adjustment of concomitant diuretics, and fluid optimization. Importantly, the incidence of significant hyperkalaemia or angioedema remains low, even in elderly or multi-morbid patients. Observational data suggest that starting at lower doses and up-titrating gradually enables most HFpEF patients - especially women and those with EF < 60% - to tolerate therapy effectively. Guideline recommendations have evolved accordingly. The 2023 ACC Expert Consensus Decision Pathway endorses ARNI use in selected HFpEF patients, particularly those with mildly reduced EF or female sex, supported by robust evidence of reduced hospitalizations and improved patient-reported outcomes [21,47].

Guideline Recommendations for HFpEF

The Contemporary European and American guidelines recognize sacubitril/valsartan as a reasonable therapeutic option to reduce HF hospitalizations in this population. These updates reflect a shift from the once-pessimistic view of HFpEF as "therapy-resistant" to one of phenotype-guided management, where ARNI occupies a central role for specific patient subsets [51].

In India and other resource-constrained regions, implementing these recommendations presents additional challenges. Cost considerations, limited awareness among general physicians, and restricted access to echocardiographic follow-up often impede timely diagnosis and initiation. Recognizing this, Indian experts have advocated for national registries and prospective outcome studies focusing on HFmrEF/HFpEF cohorts to generate local data and refine patient selection criteria. The HFSI consensus emphasizes region-specific treatment algorithms that combine ARNI with SGLT2is, lifestyle interventions, and aggressive control of comorbidities to achieve incremental benefit [47].

Expert Opinion

ARNI can be used as treatment in selected HFpEF patients, specifically those who are female, have LVEF (41-57%), have high NT-proBNP levels, and have comorbidities, such as obesity, diabetes mellitus, or hypertension, that are associated with heightened neurohormonal and oxidative stress as an adjunct to reduce the risk of HF-related hospitalizations (consensus-based, supported by subgroup analyses from PARAGON-HF). SGLT2is (Class I recommendation to reduce hospitalization) and use of diuretics to manage congestion are still the first-line therapy according to the current therapeutic hierarchy of HFpEF. ARNI is established as a Class IIb supplement for specific patients, especially when they are in the lower EF spectrum. MRAs can also be considered, especially in patients with high levels of natriuretic peptides, to maintain native renal function. Evidence suggests that ARNI has greater advantages in patients with LVEF less than 57% and is more favorable in women (subgroup analysis from PARAGON-HF; hypothesis-generating).

## Conclusions

GDMT represents a paradigm shift in HF management, with the four pillars providing complementary benefits across multiple pathophysiological pathways. Early and simultaneous initiation of these therapies is critical to optimize outcomes. In the Indian context, improving access, affordability, and implementation of GDMT remains a key priority. A structured, patient-centric approach is essential to translate evidence into real-world clinical benefit.

## Appendices

**Table 5:** Indian heart failure registries and observational studies cited in this review: Sample sizes, study populations, key GDMT and ARNI findings, and principal clinical outcomes ARNI = angiotensin receptor-neprilysin inhibitor; ACEi = angiotensin-converting enzyme inhibitor; ARB = angiotensin receptor blocker; HFrEF = heart failure with reduced ejection fraction; HFpEF = heart failure with preserved ejection fraction; HFmrEF = heart failure with mildly reduced ejection fraction; EF = ejection fraction; SBP = systolic blood pressure; eGFR = estimated glomerular filtration rate; K+ = serum potassium; BD = twice daily; CKD = chronic kidney disease; DM = diabetes mellitus; CCB = calcium channel blocker; GWTG-HF = Get With the Guidelines-Heart Failure; HFSI = Heart Failure Society of India; NT-proBNP = N-terminal pro-B-type natriuretic peptide; NNT = number needed to treat; NYHA = New York Heart Association; IHD = ischaemic heart disease; RR = rate ratio; CI = confidence interval; SZC = sodium zirconium cyclosilicate; BMI = body mass index; TTR = transthyretin; MDT = multidisciplinary team; GLP-1RA = glucagon-like peptide-1 receptor agonist; SR = strongly recommend; R = recommend

Registry/Study (Full Name)	Acronym	Sample Size (n)	Study Setting & Population	Key GDMT & ARNI Findings	Principal Clinical Outcomes & Relevance to This Review
Trivandrum Heart Failure Registry [5]	Trivandrum HFR	1,205	Multi-centre registry; 16 hospitals, Trivandrum region, Kerala. Mixed HF phenotypes (HFrEF 62%, HFmrEF 18%, HFpEF 20%); 69% male; mean age 61.2 ± 13.7 years; ischaemic heart disease most common aetiology (72%); HTN 55%, DM 52%; acute de novo HF HFrEF 36.7%, HFmrEF 44.5%, HFpEF 45.3%; consecutive admissions January-December 2013 Urban AND suburban-rural; 6% lost to follow-up	Lack of GDMT in HFrEF associated with significantly higher 5-year mortality; only 25.4% of HFrEF patients on full GDMT (BB+RAS blocker+MRA) at discharge. Discharge rates (overall cohort): BB 58%, ACE-I/ARB 48%, MRA 46%. HFrEF-specific discharge rates: BB 65.9%, ACE-I/ARB 49%, MRA 51%. ICD use: <2% despite guideline indication. Cox proportional hazards (5-year mortality): BB HR=0.64 (95% CI: 0.55-0.74, p<0.001); RAS blocker HR=0.78 (95% CI: 0.66-0.90, p=0.002); MRA HR=0.75 (95% CI: 0.64-0.88, p<0.001). ARNI: not reported (2013 cohort; pre-ARNI era in India). Frequent readmissions associated with significantly higher 5-year mortality; 5-year readmission rate 49%; 5-year mortality 69% with >1 readmission vs 50% with zero readmissions. Other independent predictors of 5-year mortality: NYHA class III/IV vs II: HR=2.8 (95% CI: 1.53-5.17, p=0.001); each 10 mmHg increase in SBP: HR=0.94 (95% CI: 0.92-0.97, p<0.001); each 1 mg/dL increase in creatinine: HR=1.11 (95% CI: 1.03-1.18, p=0.004).	5-year all-cause mortality: 59% (n=709 deaths); median survival 3.1 years; cumulative incidence rate 22.6 per 100 person-years (95% CI: 21.0-24.3). In-hospital mortality: 8.4%. 1-year mortality: 30.8%. EF-stratified 5-year mortality: HFrEF 61.3%, HFmrEF 60.5%, HFpEF 46.8%. HFpEF had 28% lower 5-year mortality vs HFrEF: HR=0.72 (95% CI: 0.57-0.92, p=0.010). Cause of death in HFrEF subgroup only: sudden cardiac death 48% (n=219) and worsening HF 48% (n=219). Cause of death in overall cohort (all 709 deaths): SCD 46%, pump failure 49%. Clinical relevance: Provides Kerala-specific evidence that GDMT underuse is directly linked to long-term mortality; only Indian registry with 5-year longitudinal follow-up and <6% attrition; despite patients being >10 years younger than Western cohorts (mean age 61.2 vs 73 years in China; 75+ in US/Europe), 5-year mortality (59%) mirrors USA (75%), Denmark (61%), Sweden (68%), UK (63%), establishing a critical GDMT optimisation target for India.
Cardiology Society of India – Kerala Acute Heart Failure Registry [6]	CSI-KHFR	7,507	Multi-centre observational registry, Kerala, 50 hospitals across all 3 regions of Kerala (north, central, south); 19 teaching and 9 government hospitals. Acute heart failure presentations; 2016 ESC criteria for AHF diagnosis; prospective follow-up at 30 and 90 days. Mean age 64.3±12.9 years; 63% male; CAD most common aetiology (65.5%); DM 61.5%; HTN 53%; de novo AHF 65% EF distribution: HFrEF: 67.5%; HFmrEF: 17.6%; HFpEF: 14.9%	Acute HF mortality independently associated with GDMT initiation; 90-day mortality risk: 43% lower with beta-blockers (HR=0.57; 95% CI: 0.47-0.70) and 40% lower with RAS blockers (HR=0.60; 95% CI: 0.48-0.76) Poor adherence to GDMT (triple therapy) documented: only 27.9% of HFrEF (n=1,415) and 20.2% of HFmrEF (n=266) received triple therapy (ACEI/ARB/ARNI + BB + aldosterone antagonist) at discharge Individual discharge GDMT rates (HFrEF): ACEI 33.2%; ARB 19.8%; beta-blocker 64.1%; Aldosterone antagonist 49.4% ARNI prescription rates - During admission: HFrEF 2.3% (n=115), HFmrEF 0.9% (n=13), HFpEF 0.3% (n=3); total across all patients 1.7% (n=131/7,507). At discharge: HFrEF 1.8% (n=52), HFmrEF 1.1% (n=7), HFpEF nil (n=0); total n=59. Low uptake attributed primarily to cost (ARNI >10× cost of ACEI/ARB) with a high discontinuation rate	Overall (pooled) mortality: In-hospital mortality: 7%; 90-day mortality: 11.6%. EF-stratified mortality: in-hospital: HFrEF 7.7%, HFmrEF 5.2%, HFpEF 5.6%; 90-day: HFrEF 12.3%, HFmrEF 9.9%, HFpEF 10.6%. 90-day rehospitalisation: overall 11.4% (HFrEF 12.1% vs HFpEF 9.4%); in-hospital VT/VF was the leading cause of death (40% of in-hospital deaths; 2.75% of total patients). Key predictors of 90-day mortality: age (HR=1.16 per decade), creatinine (HR=1.15 per 0.1 mg/dL), SBP <90 mmHg (HR=4.06), and absence of GDMT. Clinical relevance: Strongest Indian evidence that GDMT initiation is an independent predictor of short-term survival in acute HF; First EF-stratified mortality benchmarks for Indian acute HF; highlights ARNI underutilisation driven by affordability barriers in a resource-constrained LMIC setting.
National Heart Failure Registry [13]	NHFR	10,851	Pan-India multicentre registry of 53 tertiary care hospitals across 21 states and four union territories. Acute HF presentations; EF distribution: HFrEF: 65.2%; HFmrEF: 22%; HFpEF: 12.7%. Mean age: 59.9 (SD=13.5) years; 31% women; mean BMI 24.2 (SD=4.0) kg/m². Predominant aetiology: ischaemic heart disease (72%), dilated cardiomyopathy (18%), rheumatic valvular heart disease (5.9%). Co-morbidities: hypertension 48.9%, diabetes mellitus 42.3%. Recruitment: January 2019-July 2020; 74.4% de novo HF; 25.6% re-admissions.	ARNI observed in only 4.8% of cases. ARNI: HFrEF 4.8%, HFmrEF 4.7%. GDMT (triple therapy: ACEI/ARB/ARNI + BB + aldosterone blocker) received by 47.5% of eligible HFrEF patients and more than one-third of HFmrEF patients. Patients NOT receiving GDMT had significantly higher 90-day mortality in both HFrEF and HFmrEF (log-rank p<0.001 for both groups). Device therapy (CRT/ICD): 1.9% - at least 9-fold lower than high-income country rates. IV inotropes: 20.2%; non-invasive ventilation: 17.6%; invasive ventilation: 3.9%. Note: SGLT2 inhibitors were not approved at the time of enrolment in the NHFR (January 2019-July 2020) and are therefore not reported in this registry.	Overall, 90-day mortality: 14.2% (women 14.9%; men 13.9%). In-hospital mortality: 6.7% overall; EF-stratified in-hospital: HFrEF 7.5%, HFmrEF 5.1%, HFpEF 5.5%. Re-admission rate at 90 days: 8.4%. Inverse educational class–mortality gradient confirmed (p<0.001, persisted after multivariable adjustment). Key independent predictors of 90-day mortality (multivariable Cox proportional-hazards model): NYHA Class III HR=1.36 (95% CI: 1.16-1.60); NYHA Class IV HR=1.61 (95% CI: 1.37-1.90); HFpEF vs HFrEF HR=0.77 (95% CI: 0.64-0.92); serum creatinine per 1 mg/dL HR=1.09 (95% CI: 1.06-1.12); broad QRS (>120 ms) HR=1.40 (95% CI: 1.26-1.56); serum sodium <125 mEq/L HR=1.61 (95% CI: 1.30-1.98); invasive ventilation HR=5.04 (95% CI: 4.31-5.89); beta-blocker HR=0.58 (95% CI: 0.52-0.64); ACEI/ARB HR=0.83 (95% CI: 0.73-0.93); aldosterone blocker HR=0.81 (95% CI: 0.72-0.91). Clinical relevance: Largest pan-Indian HF registry (n=10,851; 53 centres; 21 states); 1 in 7 patients died within 90 days; approximately 1 in 2 eligible HFrEF patients received GDMT; GDMT adherence significantly improved survival in both HFrEF and HFmrEF. ARNI underutilisation (4.8% HFrEF, 4.7% HFmrEF) documented at national scale. SGLT2i data not applicable (not approved at enrolment).
ARNI-PRESERVED Study [33]	ARNI-PRESERVED	154	Retrospective, single-centre observational study (not a formal registry) Yashoda Hospitals, Hyderabad, Telangana, India; June-December 2020 Indian HFpEF patients (LVEF ≥50%, NYHA Class II-III, NT-proBNP ≥600 pg/mL); all patients had coronary angiography; significant CAD with complete revascularization required for inclusion Mean age 65.6±18.4 years; 56.5% female; NYHA Class III 71.4%, NYHA Class II 28.6%; DM 52.3%; HTN 78.7%; CAD 58.6%; AF 17.9%; BMI >30 kg/m² in 45.3%; 98 patients initiated ARNI in OPD; 56 initiated in-hospital prior to discharge for decompensated HF	Sacubitril/valsartan initiated at low starting doses (50 mg BD) with gradual up-titration; titrated to maximum tolerable dose and monitored in outpatient clinic. ARNI dose distribution: 50 mg BD: 24.4%; 100 mg BD: 58.8% (most common); 200 mg BD (target dose): 16.8%. Average diuretic (torsemide) dose: 10 mg. Concomitant medications: beta-blockers 92.1%; CCBs 68.7%; MRA 57.4%; diuretics 78.4%; ivabradine 44.7%; SGLT2i 15.3% Provides only India-specific real-world tolerability and efficacy data for ARNI in HFpEF cited in this review.	Clinical outcomes at 6 months: NYHA class improvement (III→II): 71.4% → 12.1% in Class III; 28.6% → 87.9% in Class II (p<0.001). Pedal oedema resolved: present in 87.6% → 10.8% (P<0.001). Rales resolved: present in 80.5% → 13.6% (p<0.001). NT-proBNP: 1607.8±304.6 → 444.3±234.9 pg/mL (p<0.001). SBP: 155.4±23.5 → 125.1±10.8 mmHg (p<0.001). DBP: 89±12.5 → 78±5.7 mmHg (p<0.001). Heart rate: 82.4±18.4 → 74±10.4 bpm (p<0.001). Echocardiographic parameters: E/A ratio: 1.4±0.3 → 1.2±0.4 (p=0.013); E/E’ ratio: 20.2±4.8 → 18.9±3.4 (p=0.006); RVSP: 38.9±15.6 → 28.3±13.3 mmHg (p<0.001); LVEF: 55.6±8.3% → 56.7±7.5% - unchanged (p=0.223); LAVI: 44.7±11.4 → 45±8.6 ml/m² - unchanged (p=0.794). Safety: Creatinine stable (1.16±0.4 → 1.14±0.3 mg/dL; p=0.62); potassium stable (4.1±0.5 → 4.2±0.4 mEq/L; p=0.053); no HF hospitalisation or deaths during 6-month follow-up. Clinical relevance: Only India-specific real-world outcome data for sacubitril/valsartan in HFpEF cited in this review; demonstrates significant symptomatic, biomarker, and diastolic functional improvement with preserved renal safety in Indian HFpEF; supports ARNI use in Indian HFpEF even in resource-limited settings.
SAVE Study [22]	SAVE	60	Retrospective cohort study, single tertiary care centre, Prayagraj, Uttar Pradesh, India Newly diagnosed adult HF patients (NYHA Class I-IV); 53.3% male (32/60), 46.7% female (28/60), mean age 54.9 ± 9.1 years; mean LVEF 34%; mean eGFR 85.03 ± 8.7 mL/min/1.73 m²; comorbidities: T2DM alone 20% (12/60), HTN alone 13.3% (8/60), both T2DM+HTN 33.3% (20/60), none 33.3% (20/60) (total with T2DM and/or HTN: 66.6%); baseline NYHA distribution (as reported in PDF Table 2; n=30 subset): Class I 20%, II 40%, III 26.7%, IV 13.3%	Sacubitril/valsartan (Sacval, Alkem Laboratories) initiated in all 60 patients; most common dose: 50 mg BD (70%), followed by 100 mg BD (25%) and 200 mg BD (5%) Significant improvement in LVEF (34%-42%; 23% relative improvement; p<0.0001) and marked reduction in NT-proBNP (1220.5-118.3 pg/mL; p<0.0001) after 3 months of treatment Comorbidities (T2DM, HTN) did not adversely affect treatment efficacy; treatment well-tolerated with <5% patients reporting hypotension	Significant improvement in LVEF (34-42%; p<0.0001) and NT-proBNP (1220.5-118.3 pg/mL; p<0.0001) at 3 months Marked improvement in NYHA functional class at 3 months: Class I 66.7% (20/30), Class II 26.7% (8/30), Class III 6.7% (2/30), Class IV 0% Significant symptom score improvements: breathlessness (6.23-2.0; p<0.0001), oedema (5.56-1.8; p<0.0001), palpitations (2.46-1.13; p<0.0001) Treatment well-tolerated; hypotension reported in <5% of patients; no patient discontinued therapy due to hypotension comorbidities (T2DM, HTN) had no adverse impact on ARNI efficacy; significant LVEF improvements across all comorbidity subgroups (p<0.0001); NT-proBNP reductions significant in T2DM-only (p=0.0429) and T2DM+HTN subgroups (p<0.0001); NT-proBNP reduction in HTN-only subgroup did not reach statistical significance (p=0.0738). Clinical relevance: Provides real-world Indian HFrEF evidence for ARNI effectiveness and safety from a North Indian tertiary centre.
iCARDIO Alliance Global Implementation Guidelines on Heart Failure 2025 [23]	iCARDIO 2025	N/A	Global guideline document by iCARDIO Alliance (International CARDIO Alliance to Improve Disease Outcomes) Writing task force: ≥50% from non-European/non-North American regions (including India, Africa, Asia, Latin America)	Strongly recommends (SR) all 4 GDMT pillars for HFrEF: SGLT2i (SR), ARNI (SR), BB (SR), MRA (SR). Strongly recommends (SR) SGLT2i + recommends (R) MRA (including finerenone (R) and spironolactone (Su)) for HFpEF. Recommends (R) ARNI (sacubitril/valsartan) for HFpEF. Suggests (Su) ACEi or ARB for HFpEF. Strongly recommends (SR) GLP-1RA (semaglutide/tirzepatide) in obese HFpEF patients Strongly recommends (SR) tafamidis/acoramidis/vutrisiran for TTR amyloid cardiomyopathy Strongly recommends (SR) IV iron (ferric carboxymaltose/ferric derisomaltose) for iron deficiency in HFrEF to improve symptoms and reduce HF hospitalisations Addresses ARNI underutilisation in LMIC including India; provides economic adjustment guidance Incorporates evidence from FINEARTS-HF, STEP-HFpEF (pooled), SUMMIT, FAIR-HF2, ATTRibute-CM, HELIOS-B, RESHAPE-HF2, TRILUMINATE, MONITOR-HF, FRESH-UP trials	Clinical relevance to this review: (1) Provides universal resource-stratified recommendations applicable to India, with explicit 'economic adjustment' guidance for 'resources somewhat limited' and 'resources severely limited' settings directly relevant to Indian HF management. (2) Confirms and reinforces GDMT underutilisation in LMIC (including India) as a major global challenge requiring systemic intervention. (3) Updates the recommendation framework to include finerenone for HFpEF/HFmrEF, GLP-1RA for obese HFpEF, and disease-modifying agents for TTR amyloidosis, addressing gaps in prior ARNI-focused review
PARAGON-HF Hypotension Sub-study [31]	PARAGON sub-study	4,796 (post hoc of PARAGON-HF RCT)	International multicentre RCT (PARAGON-HF) post hoc analysis; HFpEF/HFmrEF patients (LVEF ≥45%); age ≥50 years; NYHA class II-IV; sacubitril/valsartan vs valsartan; median follow-up ~35 months	Hypotension in 13.3% overall; 16% sacubitril/valsartan vs 11% valsartan (p<0.001). LVEF ≥60% is an independent predictor of both higher hypotension risk AND reduced clinical efficacy with sacubitril/valsartan (p=0.019). The treatment benefits of sacubitril/valsartan remained consistent regardless of the occurrence. Independent predictors of hypotension included (lack of hypertension history (HR 0.37; 95% CI: 0.29-0.48), sacubitril/valsartan assignment (HR 1.57; 95% CI: 1.34-1.84), Asian race (HR 1.76; 95% CI: 1.43-2.16), lack of diabetes history (HR 0.68; 95% CI: 0.57-0.80), higher LVEF per 5% increase (HR 1.11; 95% CI: 1.06-1.17), atrial fibrillation at randomisation (HR 1.29; 95% CI: 1.07-1.54), higher serum creatinine per mg/dL (HR 1.41; 95% CI: 1.09-1.82), MRA use at randomisation (HR 1.22; 95% CI: 1.02-1.45, previous smoking history (HR 1.59; 95% CI: 1.36-1.87; p<0.001), other race (HR 1.74; 95% CI: 1.20-2.52; p=0.004), absence of left ventricular hypertrophy on ECG (HR 0.59; 95% CI: 0.42-0.84; p=0.003), elevated NT-proBNP per doubling (HR 1.11; 95% CI: 1.03-1.19; p=0.006), paced rhythm (HR 1.45; 95% CI: 1.10-1.89; p=0.007).	Benefit/risk threshold for sacubitril/valsartan in HFpEF/HFmrEF: LVEF <60% -benefit outweighs risk (~3 primary events prevented per 1 extra hypotensive episode vs valsartan); LVEF ≥60% -no primary events prevented, ~3 extra hypotensive episodes per 100 patients. Post-hypotension outcomes: adjusted RR 1.63 (95% CI: 1.27-2.09) for CV death/total HF hospitalisation; HR 1.62 (95% CI: 1.28-2.05) for all-cause mortality; higher drug discontinuation rate (HR 1.57, 95% CI: 1.31-1.87). Clinical relevance: Provides the definitive LVEF-stratified benefit/risk framework for sacubitril/valsartan initiation in HFpEF/HFmrEF; directly applicable to Indian HFpEF patients with higher LVEF; reinforces the need for careful patient selection and hypotension monitoring. Highly relevant to Indian practice given the proportion of HFpEF patients with LVEF ≥60% in whom sacubitril/valsartan may offer unfavourable benefit/risk.
Systematic Review and Meta-Analysis of RCTs [30]	-	6,737 across 4 RCTs (PARAMOUNT, PARAGON-HF, PARALLAX, PARAGLIDE-HF); sacubitril/valsartan: n=3,375; valsartan: n=3,362	Systematic review and meta-analysis of 4 RCTs comparing sacubitril/valsartan vs valsartan exclusively in HFmrEF/HFpEF patients (LVEF ≥40-45%). PRISMA methodology; low risk of bias across all 4 studies (Cochrane RoB2). Study durations: 36 weeks (PARAMOUNT) to 4 years (PARAGON-HF). International, multicentre populations.	Efficacy outcomes: KCCQ-CSS MD +1.13 (95% CI: 0.15-2.11, p=0.024) - small but significant improvement. NYHA class improvement OR 1.32 (95% CI: 1.10-1.59, p=0.002). Composite HF hospitalisation + CV death RR 0.86 (95% CI: 0.75-0.99, p=0.04). No significant benefit for CV death (RR 0.92, 95% CI: 0.77-1.10, p=0.38) or all-cause mortality (RR 0.96, 95% CI: 0.85-1.10, p=0.56) vs valsartan. Safety outcomes: Hypotension OR 1.67 (95% CI: 1.27-2.19, p<0.0001) - significantly higher with sacubitril/valsartan. No significant increase in hyperkalaemia (OR 0.90, 95% CI: 0.78–1.03, p=0.124) or worsening renal function (OR 0.80, 95% CI: 0.55-1.16, p=0.241) vs valsartan. Sensitivity analysis excluding PARALLAX: hypotension OR 1.54 (95% CI: 1.32-1.79, p<0.0001); worsening renal function OR 0.73 (95% CI: 0.62-0.86, p=0.0001) - suggesting sacubitril/valsartan may be renoprotective.	First meta-analysis exclusively examining HFmrEF/HFpEF RCTs (vs valsartan comparator). Provides highest-level pooled evidence confirming: (1) sacubitril/valsartan significantly reduces composite HF hosp + CV death (RR 0.86) and improves NYHA class and KCCQ-CSS vs valsartan; (2) no all-cause or CV mortality benefit in HFpEF/HFmrEF; (3) hypotension is the dominant safety concern (OR 1.67) but renal function and potassium are not disproportionately worsened -directly relevant to Indian patients with CKD and T2DM in whom renal/electrolyte concerns often deter ARNI initiation; (4) sensitivity analysis suggests possible renoprotective benefit when PARALLAX removed.
Sacubitril/Valsartan Hypotension & Dosing Study [50]	Study Protocol - Results Pending	1,039 enrolled at manuscript submission (Note: data collection ongoing; final n anticipated larger)	Retrospective, multicentre cohort study. Pan-India: 160 participating centres across all major states. Indian HFrEF outpatient population (LVEF ≤40%, NYHA II-IV, aged 18-80 years). Patients initiated on sacubitril/valsartan between November 2023-May 2024. Note: This is a protocol paper. Per the published protocol: data collection to continue until the end of June 2024; analysis and interpretation of results to be completed by the end of July 2024.	Primary objective: Evaluate real-world incidence of hypotension in Indian HFrEF patients on sacubitril/valsartan and identify the best clinical practice to achieve the optimal tolerated dose (200 mg BD) without treatment discontinuation. Primary outcomes: Changes in systolic and diastolic BP (sitting, lying, standing postures); NYHA class change; % patients reaching 200 mg BD at 2 months. Secondary outcomes: Changes in HF symptoms and NT-proBNP; % patients requiring down-titration at each dose step (200 → 100 → 50 → 0 mg BD); reasons for dose reduction or discontinuation; time to reach 100 mg and 200 mg BD. Dosing protocol: 50 mg BD → 100 mg BD (at 2-4 weeks) → 200 mg BD (based on tolerance). Statistical analysis: paired t-test for BP/pulse; Wilcoxon signed-rank for NYHA class; descriptive statistics for all continuous variables.	One of the largest Indian real-world studies of sacubitril/valsartan in HFrEF. Clinical relevance: (1) Addresses the principal barrier to optimal ARNI dosing in India - hypotension - in a pan-India, real-world outpatient setting across 160 centres, providing the most geographically representative Indian ARNI dataset to date; (2) Will quantify exact hypotension incidence rates and dose down-titration frequencies in Indian HFrEF practice, directly informing recommendations on starting doses and titration intervals; (3) The study design (retrospective real-world HFrEF, 160 centres, 19 investigators across 11 states/UTs) represents the ‘large real-world Indian dataset’. (4) Results will provide India-specific benchmarks for sacubitril/valsartan tolerability that are currently extrapolated from non-Indian populations. Ongoing study (results pending): The current study has published the protocol for a large-scale retrospective, multicentre real-world cohort study assessing hypotension incidence and sacubitril/valsartan dosing strategies in Indian HFrEF patients across 160 centres nationwide (n=1,039 enrolled at protocol publication; final cohort larger).
